# Macrobicyclic Dibridgehead Di(trialkyl)pnictogens E((CH_2_)_*n*_)_3_E (E/*n* = As/10, As/12, As/14, Sb/14) and Their Cage-like Metal Complexes: Syntheses, Structures, and Homeomorphic Isomerizations

**DOI:** 10.1021/acs.organomet.4c00120

**Published:** 2024-05-21

**Authors:** Samuel R. Zarcone, Peter J. Verardi, Gong M. Chu, Nattamai Bhuvanesh, John A. Gladysz

**Affiliations:** Department of Chemistry, Texas A&M University, PO Box 30012, College Station, Texas 77842-3012, United States

## Abstract

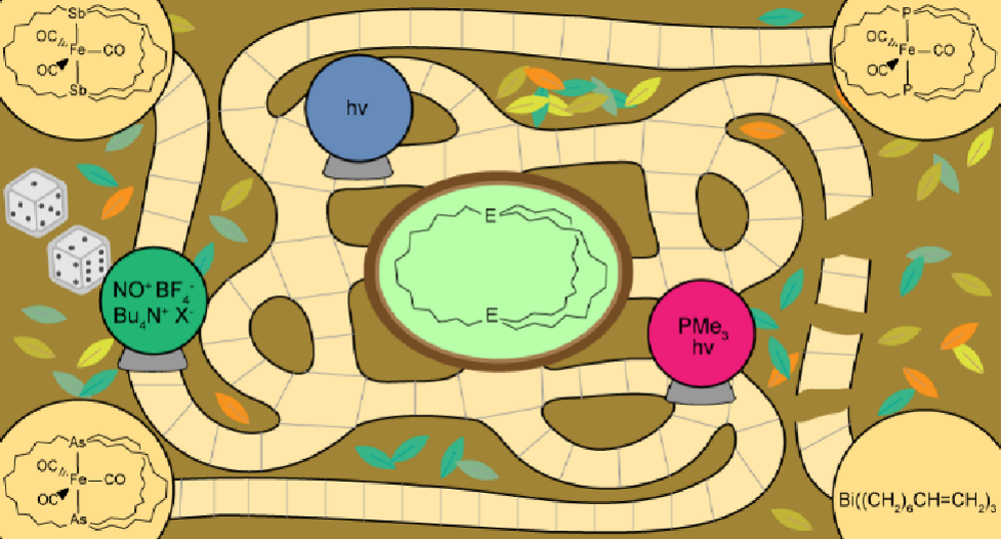

Photolyses of *trans*-Fe(CO)_3_(As((CH_2_)_*n*_)_3_As) (*n* = **a**, 10; **b**, 12; **c**, 14) in the presence of PMe_3_, or reactions of *trans*-[Fe(CO)_2_(NO)(As((CH_2_)_*n*_)_3_As)]^+^ BF_4_^–^ and *n*-Bu_4_N^+^ Cl^–^, afford the air stable title complexes As((CH_2_)_*n*_)_3_As (**8a**–**c**) in 79–34% yields. With **8a**, the *in*, *in* and *out*, *out* isomers are separable and each is crystallographically characterized. With **8b**,**c**, the isomers rapidly interconvert by homeomorphic isomerization, but each crystallizes (contrathermodynamically) as an *out*, *out* isomer. Reactions of **8c** with H_2_O_2_ or **8b** with BH_3_ give **8c**·2O or **8b**·2BH_3_, respectively (85–94%). Reactions of **8c** with MCl_2_ (M = Pt, Pd, Ni), Rh(CO)(Cl), and Fe(CO)_3_ sources afford the corresponding cage-like complexes *trans*-ML_*n*_(As((CH_2_)_14_)_3_As) (86–51%). The crystal structures of **8c**·2O and the PtCl_2_ and PdCl_2_ adducts are determined and compared to those of **8a**–**c** and diphosphorus analogs. The corresponding distibine Sb((CH_2_)_14_)_3_Sb is analogously prepared, but precursors necessary for the bismuth analog could not be accessed due to the diminished BiR_3_ Lewis basicity.

## Introduction

**Scheme 1:**
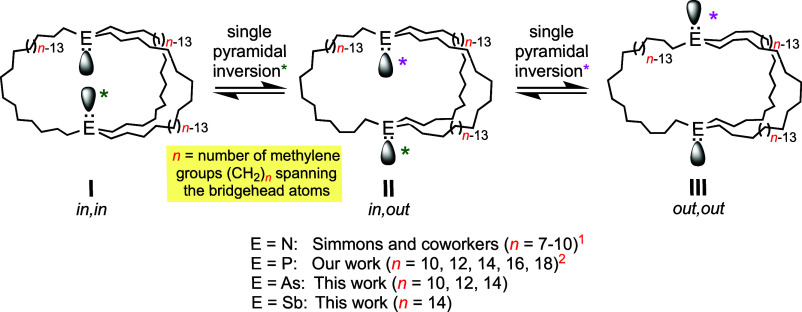
Interconversions of *in*, *in* (**I**), *in*, *out* (**II**), and *out*, *out* (**III**) Isomers of E((CH_2_)_*n*_)_3_E by Pyramidal Inversion of the Bridgehead Atoms E

Macrobicyclic dibridgehead di(trialkyl)pnictogens of the formula E((CH_2_)_*n*_)_3_E have been surprisingly little studied, despite their fundamental position in the hierarchy of bicyclic organic molecules and increasing recognition of their unusual properties.^1,2^ As illustrated by **I**–**III** in Scheme 1, they are capable of *in*/*out* stereoisomerism.^3^ These species can interconvert by pyramidal inversion (epimerization) of the bridgehead pnictogen atoms, which is facile for nitrogen at room temperature, but requires heating to ca. 150 °C for phosphorus and ≥200 °C for arsenic and antimony.^4^

**Scheme 2:**
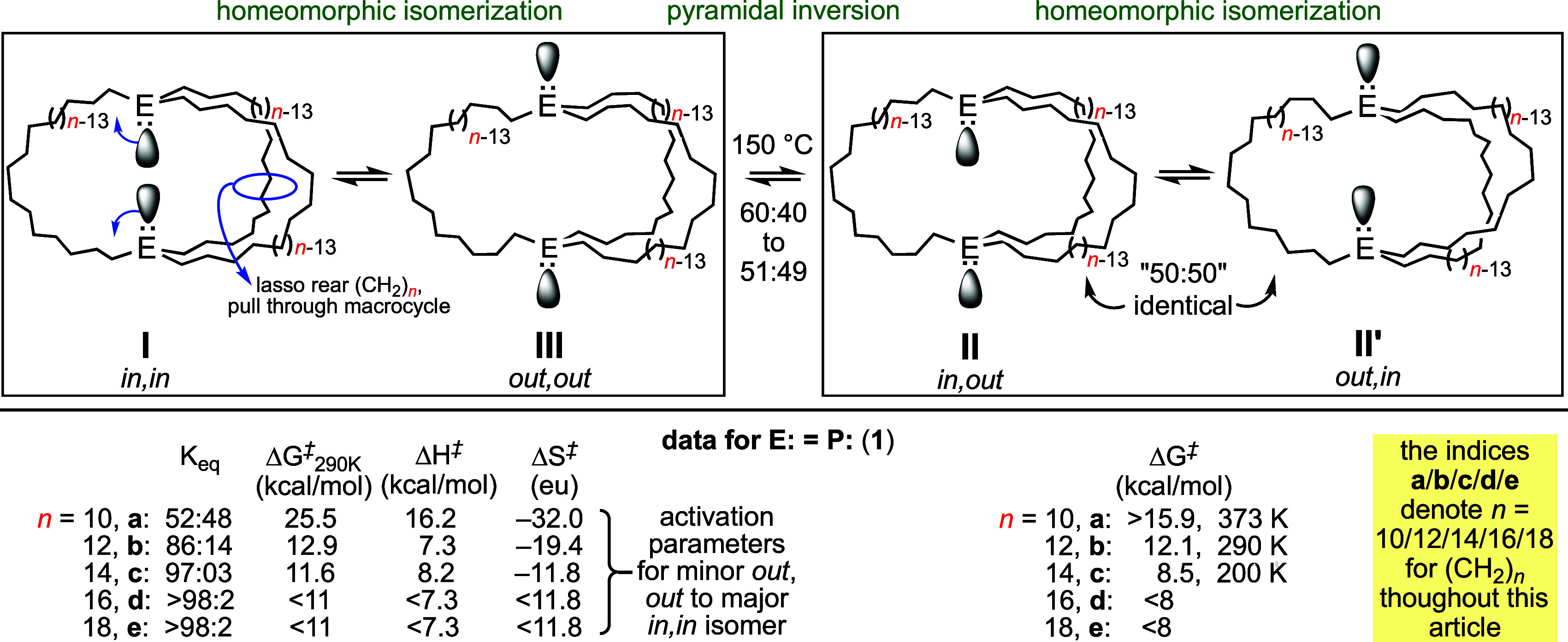
Interconversions of *in*, *in* (**I**) and *out*, *out* (**III**) Isomers, and Degenerate *in, out* (**II**) and *out, in* (**II′**) Isomers, by Homeomorphic Isomerizations

It is much less widely appreciated that the *in*, *in* and *out*, *out* isomers **I** and **III**—as well as the two degenerate forms of **II** (*in, out* and *out, in*)—can also interconvert by a topological process as sketched in Scheme 2. To our knowledge, this pathway was first conceptualized by H. E. Simmons (duPont),^1a,1b^ who reported the series of dibridgehead diamines N((CH_2_)_*n*_)_3_N (*n* = 7–10) and the corresponding ammonium salts [HN((CH_2_)_*n*_)_3_NH]^2+^ 2X^–^ in 1968. Higher homologues, up to *n* = 34, were treated in a subsequent patent.^1c^ He pointed out that both types of molecules could essentially turn themselves inside out, which he termed homeomorphic isomerization. This can be visualized as “pulling” one E(CH_2_)_*n*_E segment through the macrocycle defined by the other two, as depicted in Scheme 2 and a video.^2b^

In actuality, Simmons’ data did not provide any direct support for this pathway. In the case of N((CH_2_)_*n*_)_3_N, the three isomers interconverted too rapidly to be individually detectable. With the diprotonated salts [HN((CH_2_)_*n*_)_3_NH]^2+^ 2X^–^ examined, *in/out* isomerization was shown to entail nitrogen deprotonation, pyramidal inversion, and reprotonation. However, a few other groups have been able to unequivocally demonstrate the operation of homeomorphic isomerization in macrobicyclic compounds with different types of bridgeheads and linkers.^5^

**Scheme 3:**
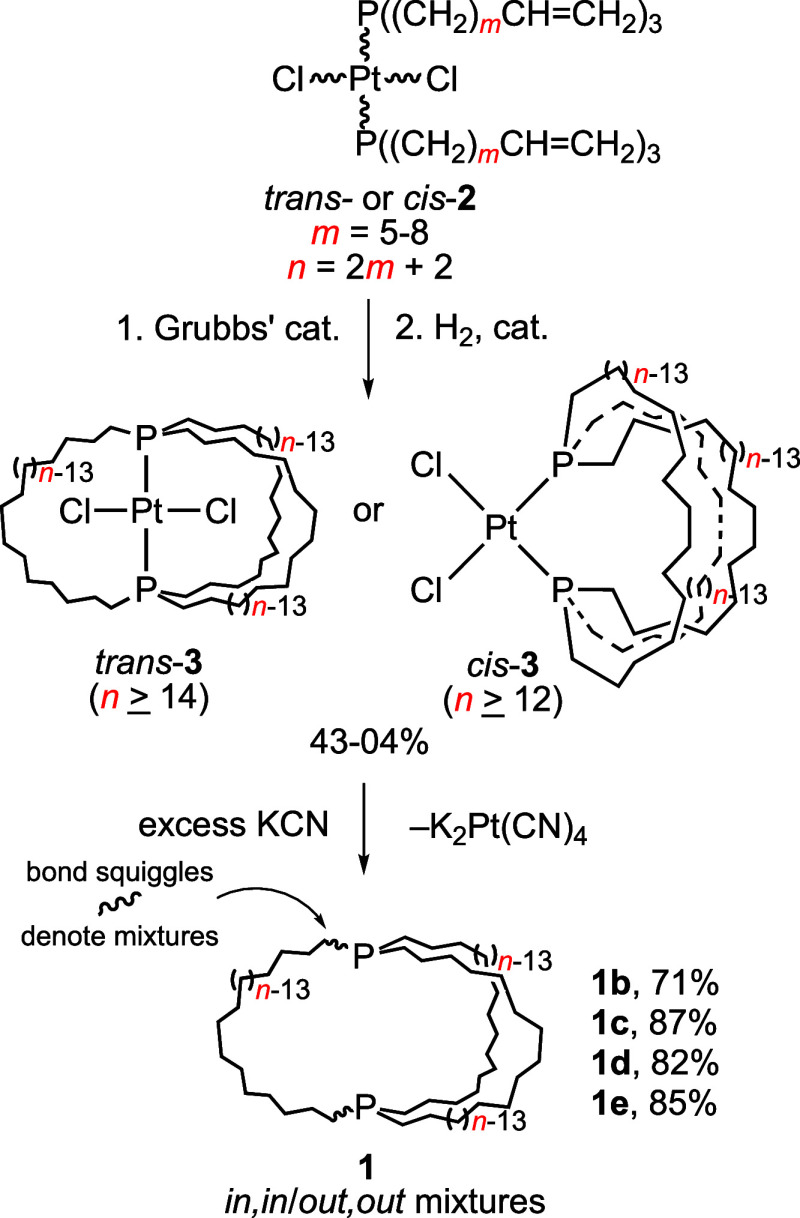
Syntheses of Macrobicyclic Dibridgehead Diphosphines P((CH_2_)_*n*_)_3_P (**1**) via Platinum Complexes

**Scheme 4:**
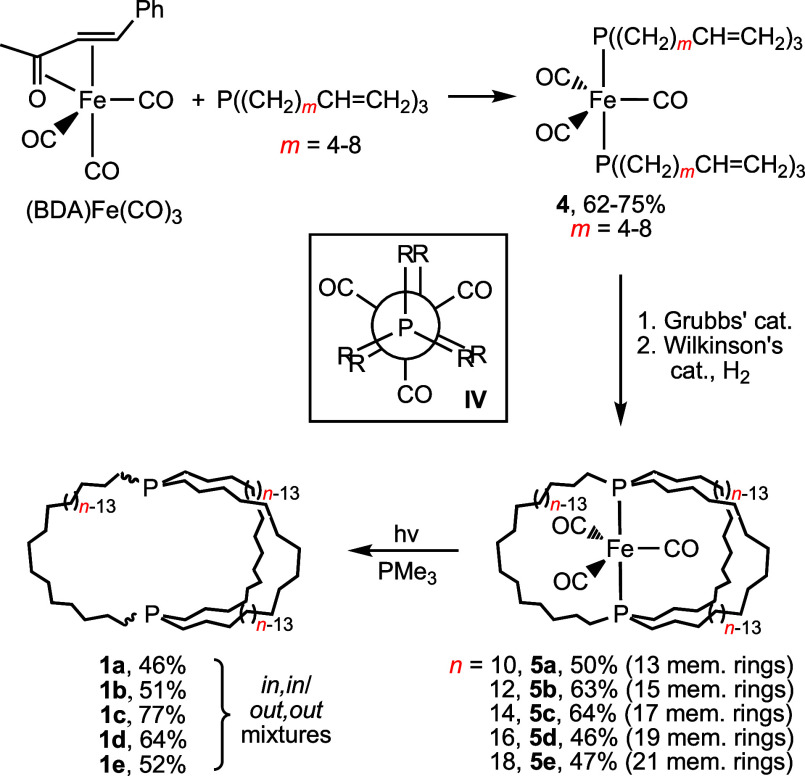
Syntheses of Macrobicyclic Dibridgehead Diphosphines P((CH_2_)_*n*_)_3_P (**1**) via Iron Complexes

We entered this field via serendipitous syntheses of the dibridgehead diphosphines P((CH_2_)_*n*_)_3_P (**1**), first by the platinum-based pathways shown in Scheme 3.^2a^ Previously, the largest such bicyclic compounds featured short P(CH_2_)_4_P linkages.^6^ More recently, the less costly and more general and efficient iron-based route in Scheme 4 has been developed.^2c^ Both pathways feature 3-fold *intra*molecular and *inter*ligand ring-closing olefin metatheses. However, the acyclic trigonal bipyramidal iron intermediates **4**, but not the square planar platinum counterparts *trans*-**2**, are able to adopt the doubly staggered P–Fe–P conformation **IV**. This is preorganized to facilitate intramolecular ring-closing as opposed to oligomerization. Due to the much higher barriers to bridgehead atom inversion in **1** versus nitrogen homologues, it has proved feasible to establish rates and equilibria for all possible isomerization pathways. Key data are summarized in Scheme 2 (bottom).^2c^

We next sought to study analogous arsenic, antimony, and bismuth compounds. There are many well established group 15 periodic trends,^7,8^ but we were particularly motivated by the progressively longer arsenic–metal, antimony–metal, and bismuth–metal bonds.^9^ It was thought that this might lead to lower ML_*y*_ rotational barriers about the E-M-E axes in heavier analogs of **2** and **5**, which are often termed “gyroscope like”.^10−13^ In earlier work we had found that upon going from phosphorus to arsenic, barriers could drop by ∼3 kcal/mol.^13^ Furthermore, the crystal lattices exhibited by species of the formula *trans*-Fe(CO)(NO)(X)(P((CH_2_)_14_)_3_P) (X = halide or cyanide) have several features that render such adducts attractive for functional molecular gyroscopes, as elaborated elsewhere.^10^

**Figure 1:**
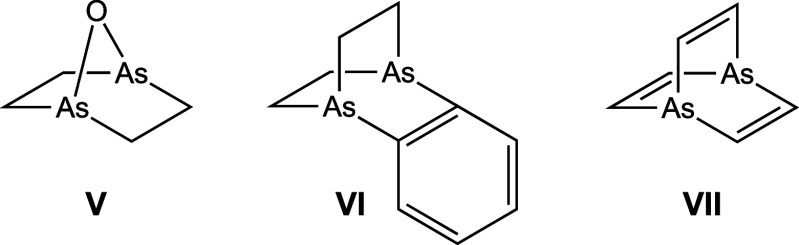
Largest dibridgehead diorganoarsines with at least two As(CH_2_)_*n*_As units (**V**, **VI**), and a CH=CH bridged barrelene-type system (**VII**).

Surprisingly, prior to this work dibridgehead diarsines As((CH_2_)_*n*_)_3_As were unknown for all values of *n*, although as shown in Figure 1 compounds with two short As(CH_2_)_2_As segments and AsOAs (**V**) or As(C=C)As (**VI**) bridges had been isolated.^14^ Dibridgehead diarsines with three CH=CH bridges have been reported (**VII**),^15^ as well as tris(benzannulated) analogs for all pnictogens.^16^ Macrobicyclic systems in which each arsenic atom features three thiolate linkages, As(SCH_2_(arylene)CH_2_S)_3_As, are described below.^17^

**Scheme 5:**
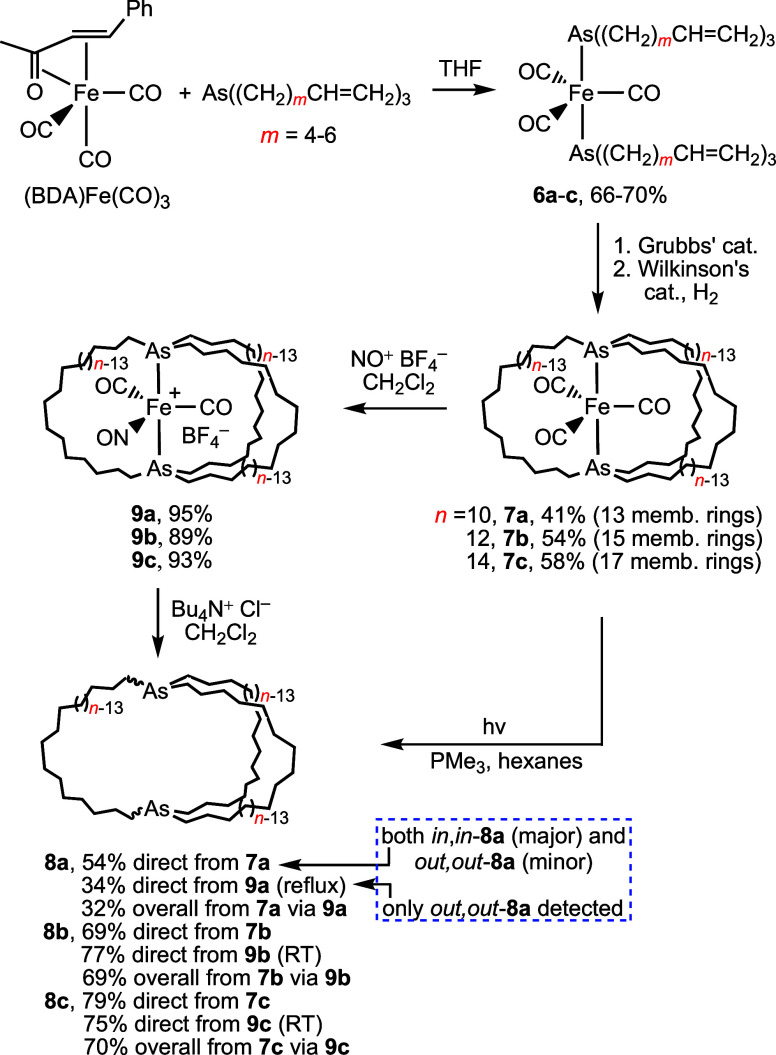
Syntheses of Macrobicyclic Dibridgehead Diarsines As((CH_2_)_*n*_)_3_As (**8**)

As sketched in Scheme 5, we had prepared several diarsine complexes *trans*-Fe(CO)_3_(As((CH_2_)_*n*_)_3_As) (**7**) analogously to the diphosphine complexes **5** (Scheme 4). In this paper we show that **7** provide excellent precursors to free dibridgehead diarsines As((CH_2_)_*n*_)_3_As (**8**). Exploratory studies with antimony analogs are also described. These diarsines can, per their parentage, serve as cage-like *trans*-spanning ligands for a variety of metal fragments, and readily undergo other types of derivatization. A small portion of this work has been communicated.^18^

## Results

### Syntheses of Dibridgehead Diarsines

As reported earlier, reactions of (BDA)Fe(CO)_3_ and the readily available arsines As((CH_2_)_*m*_CH=CH_2_)_3_ (*m* = 4–6) give the adducts *trans*-Fe(CO)_3_(As((CH_2_)_*m*_CH=CH_2_)_3_)_2_ (**6a**−**c**) in 66–70% yields (Scheme 5).^13^ These were treated with Grubbs’ catalyst and the resulting crude tris(alkenes) hydrogenated using Wilkinson’s catalyst, giving **7a**−**c** in 41–58% yields. The macrocycle sizes ranged from 13 to 17 atoms, with the former representing the lower limit for the diphosphine chemistry in Scheme 4. Since arsenic–carbon bonds are longer than phosphorus–carbon bonds, we wondered whether the next lower homologue of **6a**, *trans*-Fe(CO)_3_(As((CH_2_)_3_CH=CH_2_)_3_)_2_, might allow access to the next lower homologue of **7a**, with 11 membered rings. The preparation of this new complex is described in the Supporting Information (SI). However, under all conditions investigated, metathesis was sluggish, and no well-defined products could be detected.

Next, **7a**−**c** were treated with NO^+^ BF_4_^–^ as described earlier to give the cationic dicarbonyl complexes *trans*-[Fe(CO)_2_(NO)(As((CH_2_)_*n*_)_3_As)]^+^ BF_4_^–^ (**9a**–**c**; 95–93%). The original goal was to replace a CO ligand with a halide or pseudohalide ligand (X) to afford complexes of the formula *trans*-Fe(CO)(NO)(X)(As((CH_2_)_*n*_)_3_As), a process easily carried out with the diphosphorus analogs.^12b^ However, extensive efforts involving a large catalog of nucleophiles (KCN; PNP^+^ Cl^–^; *n*-Bu_4_N^+^ X^–^ with X = Br, I, CN, OCl; NaC≡CH) and conditions (THF/acetone/neat, –35 °C to RT) were unsuccessful. Solutions became dark green, but IR spectra did not reveal any ν_CO_ or ν_NO_ bands and the iron byproducts remain unidentified.

On the positive side, in nearly every case a dibridgehead diarsine As((CH_2_)_*n*_)_3_As (**8a**–**c**) was produced, and the best preparative conditions (*n*-Bu_4_N^+^ Cl^–^, CH_2_Cl_2_) afforded isolated yields of 77–34%. Apparently, the weaker metal–arsenic versus metal–phosphorus bonds^19^ facilitate ligand displacement, leading to orthogonal reactivity channels for **9a**–**c** and the diphosphine analogs. For the formation of the smallest diarsine macrobicycle **8a**, higher temperatures were required (refluxing CH_2_Cl_2_ vs room temperature).

Somewhat later, the photolytic route to the diphosphines **1** in Scheme 4 was discovered.^2c^ As shown in Scheme 5, it was then found that irradiation of hexane solutions of **7a**−**c** containing PMe_3_ (10 equiv) with a Hanovia 450 W lamp similarly afforded the diarsines **8a**−**c** in 79–54% yields. This enables two steps to be replaced by one, in most cases in higher overall yields. In the absence of PMe_3_, yields of **8c** were only slightly reduced, but those of **8a** dropped to <10% (26% in CH_2_Cl_2_; 12% in toluene).

Unlike all other samples, the ^13^C{^1^H} NMR spectra of **8a** produced photochemically gave two sets signals. Two isomers with similar but distinct spectra could be isolated in 41% and 13% yields after passage through silica gel. As elaborated below, these could be assigned as *in,in*-**8a** (dominant photochemically) and *out,out*-**8a** (minor photochemically but sole isomer from **9a** and Cl^–^). The larger macrocycles **8b**,**c** are of course also capable of *in/out* isomerism, but like their phosphorus analogs^2^ were expected to rapidly interconvert on the NMR time scale. Accordingly, **8b** gave only a single set of signals in toluene-*d*_8_ at −80 °C.^20^

Compounds **8a**−**c** were obtained as air stable, analytically pure white solids. Most ^13^C{^1^H} signals could be assigned based upon an extensive earlier study of acyclic symmetrical diarsines Ph_2_As(CH_2_)_*n*_AsPh_2_ (*n* = 6–12, 16).^21^ However, since ^75^As (natural abundance 100%) has a nuclear spin (*I*) of 3/2 instead of 1/2, many analyses that could be carried out with the diphosphines **1** were precluded. Compounds **8a**−**c** underwent decomposition starting at ∼150 °C, blocking entry to the *in, out* isomer manifold that could be accessed with the diphosphines **1** (Scheme 2).

When toluene-*d*_8_ solutions of *in,in*-**8a** were kept at 80 °C for 1 h, ^13^C{^1^H} NMR spectra showed a ca. 50:50 mixture of *in*, *in* and *out*, *out* isomers. This ratio did not change after an additional 186 h. An identical mixture was obtained when toluene-*d*_8_ solutions of *in,in*-**8a** were kept at room temperature for 240 h. Thus, the equilibrium ratio is close to that of the diphosphine analog **1a** in Scheme 2 (48:52 *in,in*/*out,out*). Isomerizations of *in*,*in*-**1a** were somewhat slower than those of *in,in*-**8a**, as judged from solutions kept at room temperature.

### Crystal Structures of Dibridgehead Diarsines

For the dibridgehead diphosphines **1a**−**e** (Scheme 4), there is excellent evidence that *in*, *in* isomers are more stable than *out*, *out* isomers in solution. Among other factors, dispersion forces have been invoked.^2b,2c^ However, for the smallest macrobicycle **1a** the ratio is close to 50:50 (Scheme 2).^2c^ For much smaller bicycles such as the diarsines **V**-**VII** in Figure 1, *out*, *out* isomers should greatly dominate for obvious steric reasons. In any case, **1a**,**b**,**c**,**e** crystallize contrathermodynamically, as *out*, *out* isomers.^2^ As reflected by the activation parameters in Scheme 2, homeomorphic isomerization is, except for **1a**, rapid under most crystallization conditions.

**Figure 2:**
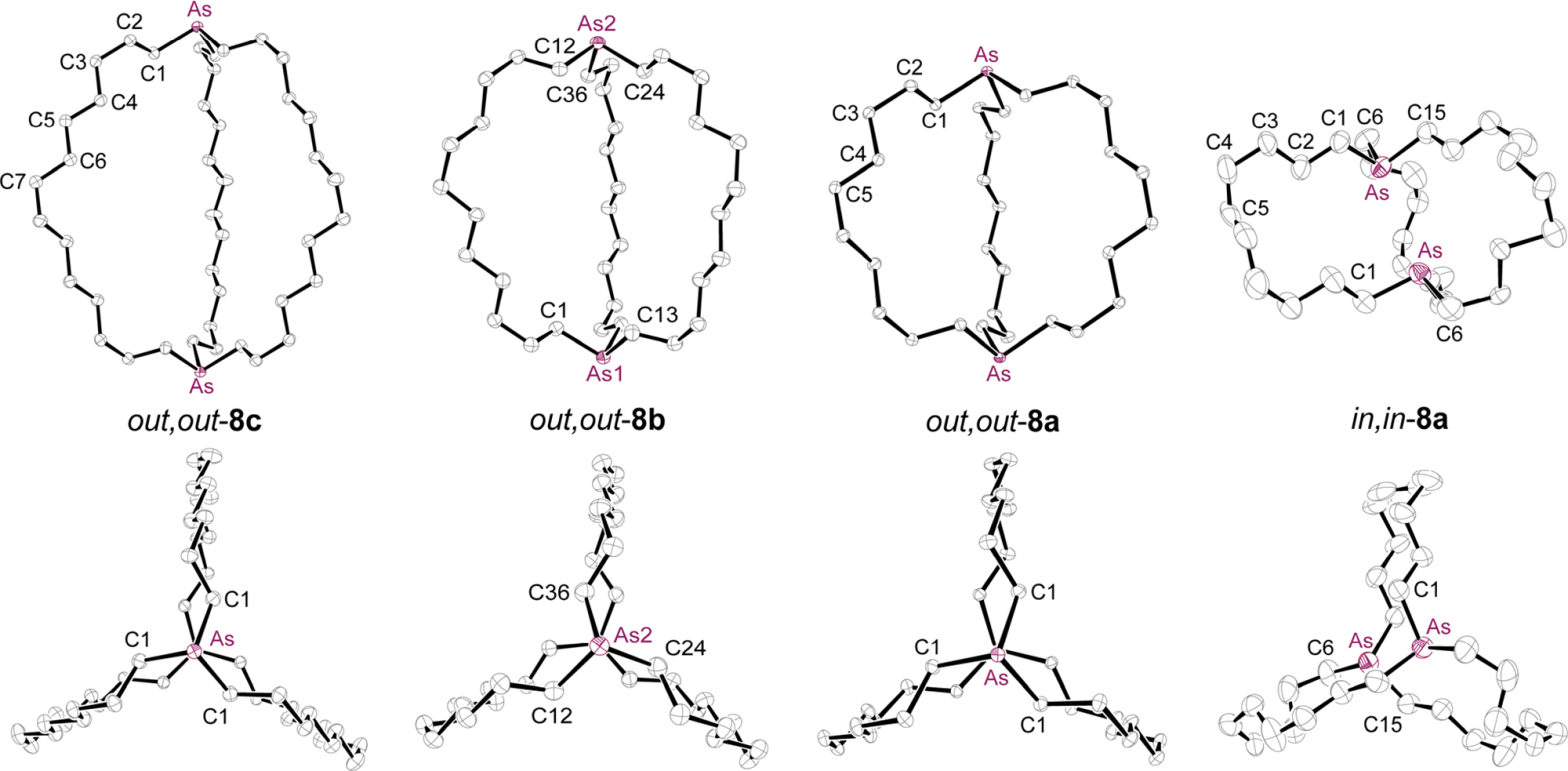
Thermal ellipsoid diagrams (50% probability) of the molecular structures of diarsines **8** with hydrogen atoms omitted (for the seven disordered CH_2_ groups *in,in*-**8a**, only the dominant conformation is depicted).

Crystals of the corresponding diarsines **8a** (both isomers), **8b**, and **8c** were grown as described in the experimental section. Their X-ray structures were determined as summarized in the SI and Table S1. Key metrical parameters are provided in Table 1, and thermal ellipsoid diagrams are depicted in Figure 2.

**Table 1:** Key Crystallographic Distances [Å] and Angles [°] for Dibridgehead Diarsines and Diarsine Dioxides, Including Selected Data for the Phosphorus Analogs *out,out*-**1a**,**b**,**c**

	*in,in*-**8a**	*out,out-***8a**	*out,out*-**8b**	*out,out*-**8c**	*out,out-***8c**·2O·4(H_2_O)
As···As	4.4834(4)	8.8954(4)	11.145(1)	13.2873(15)	16.771(1)
P···P^a^	–	8.6136(14)	10.8271(8)	12.948(3)	–
As–C	1.968(3)	1.9710(12)	1.9798(17)	1.9754(17) 1.9754(17)	1.951(6)
	1.988(3)		1.9765(17)	1.9753(17)	1.928(6)
	1.975(3)		1.9746(18)		1.931(6)
			1.9813(18)		1.948(6)
			1.9755(18)		1.925(6)
			1.9773(17)		1.909(6)
As–O	–	–	–	–	1.676(4)
					1.669(4)
C–As–C	98.25(13)	95.96(5)	95.24(7)	95.91(7)	106.1(3)
	97.23(12)		97.10(8)		113.0(2)
	98.26(14)		95.55(8)		112.2(3)
			96.61(7)		113.6(3)
			95.37(7)		106.1(3)
			96.32(7)		108.4(3)
C–As–LP^b^ or C–As–O	119.44	120.9	120.9	121.0	112.3(2)
	119.40		120.8		107.6(2)
					105.6(2)
					109.2(2)
					105.8(2)
					113.9(2)
As···As–LP^b^ or As···As–O	40.4	180.0	173.4	180.0	167.1(1)
					150.9(1)
arsenic pyramidalization^c^	293.7	287.9	287.9	287.7	331.3
			288.3		328.1
P···P-lone pair^a^^,^^b^	–	180.0	172.9	180.0	–
			173.5		
phosphorus pyramidalization^a^^,^^c^	–	295.2	295.4	295.9	–
			295.0		

a Data for *out*,*out*-**1a**,**b**,**c**.

b Angles involving lone pair are derived from those of hydrogen atoms introduced in geometrically idealized positions.

c Sum of the three C-E-C bond angles (for limiting values, see text).

As shown in Figure 2 (left column), the largest macrobicycle, **8c**, crystallizes as an *out*, *out* isomer. It exhibits remarkably high molecular symmetry, with a *C*_3_ axis passing through the two arsenic atoms and three *C*_2_ axes in a perpendicular plane, corresponding to the point group *D*_3_, and furthermore occupies a special position with *D*_3_ crystallographic site symmetry. Nonetheless, the molecule remains chiral, and both enantiomers are present in the unit cell. The two As···As–LP angles are 180°, as would be expected of an idealized *out*, *out* structure.

As depicted in the second column of Figure 2, the intermediately sized macrobicycle **8b** also crystallizes as an *out*, *out* isomer. The conformation is similar to that of *out,out*-**8a**, but there is no molecular symmetry. The two As···As–LP angles are slightly less than 180° (172.4°, 172.8°; Table 1), and the arsenic–arsenic distance is shorter than that of *out,out*-**8c** (11.145(1) Å vs 13.2873(15) Å).

The next two structures establish the stereochemistry of the chromatographically separated isomers of **8a**. As shown in the third column of Figure 2, *out,out*-**8a** provides a second example of *D*_3_ molecular (or crystallographic *D*_3_ site) symmetry, but with the arsenic–arsenic distance (8.8954(4) Å) much shorter than those of *out,out*-**8b**,**c**. Interestingly, crystalline *out,out*-**8a**−**c** are isostructural with the diphosphorus analogs, although the arsenic–arsenic distances are ca. 3% longer than the phosphorus–phosphorus distances (8.6136(14), 10.8271(8), 12.948(3) Å; Table 1).

As shown in the right column of Figure 2, the macrobicycle of *in,in*-**8a** skews somewhat, presumably to increase spacing between the bridgehead arsenic atoms. However, the separation (4.4834(4) Å) is much greater than twice the van der Waals radius of arsenic (2 × 1.85 Å).^22^ This distortion increases the two As···As–LP angles, which would be 0° in an idealized *in*, *in* geometry, to 40.4°. Similar skewing occurs in the corresponding diphosphorus compound in which the arsenic atoms have been replaced by *in*, *in* PBH_3_ units,^2c^ and Lewis acid adducts of *in, out* isomers of **1c**.^23^ The molecular structure of *in,in*-**8a** also exhibits a *C*_2_ symmetry axis that passes through the midpoint of the two bridgehead arsenic atoms and the center of the C5–C5′ bond.

**Figure 3:**
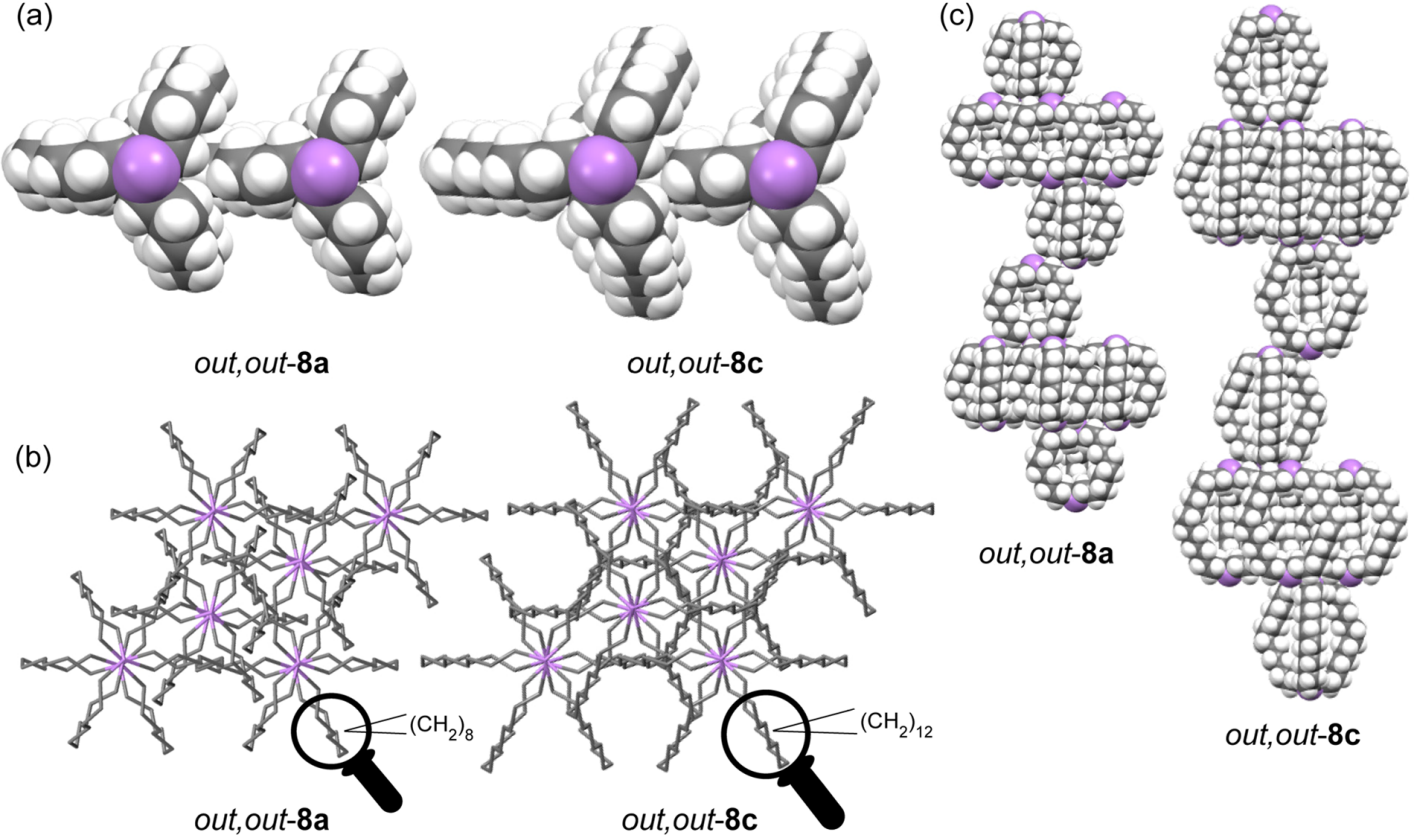
Selected outtakes of the crystal lattices of diarsines *out,out*-**8a** and *out,out*-**8c** with the long *c* axis perpendicular to the plane of the paper (a, b) or in the plane of the paper (c).

To better understand the marked preference for the diarsines **8** and diphosphines **1** to crystallize as *out*, *out* isomers, their lattices have been analyzed in detail. Figures 3a and 3b represent views down the long *c* axes of *out,out*-**8a**,**c** (63.279(3) and 90.185(10) Å; Table S1). These feature parallel stacks of arsenic atoms (purple). As shown in Figure 3a, the (CH_2_)_*n*_ bridge of one molecule of *out,out*-**8a**,**c** intercalates between two (CH_2_)_*n*_ bridges of an adjacent molecule, a motif not available to skewed variants such as *in*,*in*-**8a**. As depicted in Figure 3b, each stack is surrounded by six equidistant stacks. From each emanates six apparent rhomboids derived from two AsCH_2_ linkages, followed by tails representing (CH_2_)_8_ or (CH_2_)_12_ segments. Three rhomboids are associated with one set of molecules in the stack, and the other three with a second set.

Figure 3c, in which the long *c* axis is now in the plane of the paper, shows how the molecules associated with each parallel stack of arsenic atoms are segregated into layers (the six rhomboids originate from molecules three layers apart). The layer–layer spacings are greater for *out,out*-**8c** than *out,out*-**8a** (15.03 vs 10.55 Å), matching the ratios of the *c* axes. However, the distances between the parallel stacks (Figure 3b) are similar (8.019 vs 8.045 Å). Hence, the four additional carbon atoms in each methylene chain of *out,out*-**8c** render the molecule “taller” but not “fatter”.

Thus, while a number a special features can be identified in the crystal lattices of certain *out*, *out* isomers, these fall short of rigorous rationales for their preferential crystallization. In the analysis of polymorphs, the denser modification is often found to be the more stable.^24^ When the unit cell volumes of *in,in*-**8a** and *out,out*-**8a** are normalized to the number of molecules (*Z*), the former is found to be ca. 2% more compact (775.16 vs 788.16 Å^3^). However, a lower melting point (53–57 °C vs 112–114 °C for analytical samples) argues for weaker lattice interactions.

Besides thermodynamics, there is also a kinetic question. The crystals of *in,in*-**8a** analyzed were obtained from a chromatographically purified sample at −40 °C (see SI). When crystals were grown from a sample of *in,in*-**8a** in MeOH/CH_2_Cl_2_ at room temperature, where a ca. 50:50 equilibrium mixture can eventually be realized, only *out,out*-**8a** was obtained. Mixtures of both isomers resulted at intermediate temperatures.^25^ Hence, *out,out*-**8a** crystallizes much more rapidly from *in,in*/*out*,*out* mixtures than *in,in*-**8a**.

### Dibridgehead Diarsine Chemistry

**Scheme 6:**
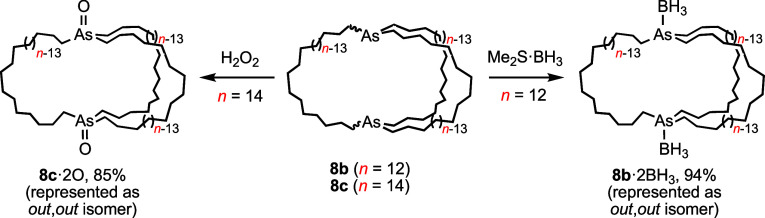
Main Group Adducts of Diarsines **8b**,**c**

The dibridgehead diphosphines **1a**−**e** can be cleanly converted to oxides and borane adducts,^2^ as can many trialkylarsines.^26,27^ Thus, **8c** was treated with excess H_2_O_2_ as shown in Scheme 6. Workup gave the expected dibridgehead diarsine dioxide OAs((CH_2_)_14_)_3_AsO (**8c**·2O) as a hygroscopic white solid in 86% yield. An IR spectrum showed a characteristic ν_As=O_ band at 881 cm^–1^.^26,28^ A reaction of **8b** and Me_2_S·BH_3_ (∼3 equiv/As) afforded the oily bis(borane) H_3_BAs((CH_2_)_12_)_3_AsBH_3_ (**8b**·2BH_3_) in 94% yield. Per previous experience with the diphosphine diboranes **1a**−**c**·2BH_3_, only a single set of ^13^C{^1^H} signals was observed when spectra were recorded at −80 °C in CD_2_Cl_2_. Although the dominant isomer in solution cannot be assigned with certainty, it is believed to be *out*, *out* as depicted in Scheme 6.

**Figure 4:**
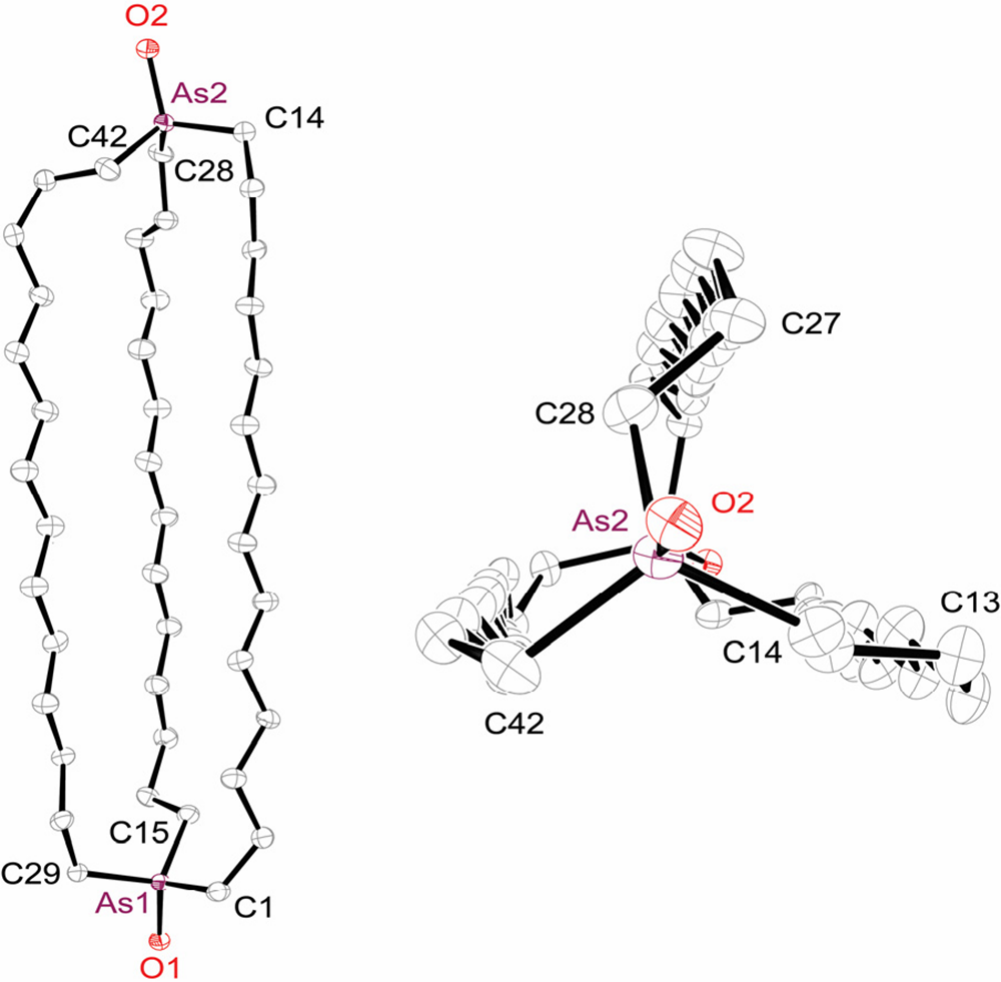
Thermal ellipsoid diagrams (50% probability) of the diarsine dioxide **8c**·2O·(H_2_O)_4_ with hydrogen atoms and H_2_O molecules omitted.

The dioxide **8c**·2O could be crystallized, and an X-ray structure (Figure 4) showed an *out*, *out* isomer. Key metrical parameters are given in Table 1. The bicyclic core was much more distorted than in *out,out*-**8a**−**c**, with (1) As···As–O angles (150.9°, 167.1°) significantly lower than the idealized 180°, (2) an As···As distance much greater than in the precursor *out,out*-**8c** (16.771(1) Å vs 13.2873(15) Å), and (3) no symmetry elements. Arsine oxides are good hydrogen bond acceptors,^29^ and four water molecules were present for every molecule of **8c**·2O. However, the data did not suffice for further analyses. The arsenic–oxygen bond lengths (1.676(4), 1.669(4) Å) were close to those found in anhydrous and monohydrated triphenylarsine oxide (1.65 Å and 1.644(7)–1.657 Å, respectively).^30^ Crystal structures of diphosphine dioxides of **1a**−**e** are not yet available, but those of some less symmetrical dibridgehead species are.^31^

**Scheme 7:**
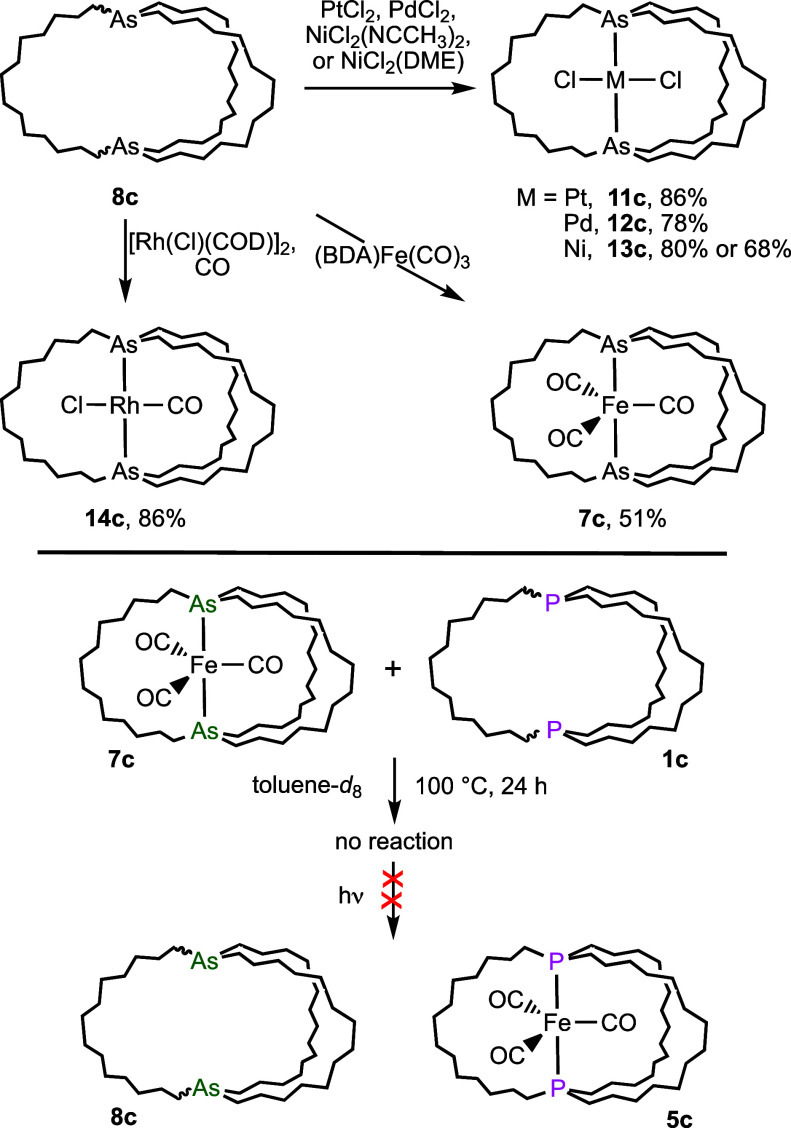
Transition Metal Adducts of Diarsine **8c** and Attempted Diarsine/Diphosphine Exchange

Several reactions of diphosphines **1** with functional equivalents of metal fragments ML_*y*_ have been described.^2b,32,33^ In favorable cases, gyroscope-like complexes *trans*-ML_*y*_(P((CH_2_)_*n*_)_3_P) result. Thus, the diarsine **8c** and PtCl_2_ were combined in toluene. As shown in Scheme 7, workup gave *trans*-Pt(Cl)_2_(As((CH_2_)_14_)_3_As) (**11c**) in 86% yield as a pale yellow solid. Combining **8c** with PdCl_2_ or NiCl_2_ sources gave the pale yellow palladium and intensely purple nickel analogs **12c** and **13c** (80–68%). Diphosphine analogs of **11c**−**13c** have been previously reported (e.g., *trans*-**3c** in Scheme 3).^11,32−34^ All new complexes were characterized by NMR and microanalysis. When ^13^C{^1^H} NMR spectra of **11c** and **12c** were recorded in CD_2_Cl_2_ at −80 °C, only a single set of seven ^13^C{^1^H} NMR signals was observed, indicative of rapid MCl_2_ rotation on the NMR time scale.^34^

Similar reactions of **8c** with [Rh(Cl)(COD)]_2_/CO and (BDA)Fe(CO)_3_ were investigated. As shown in Scheme 7, the former afforded *trans*-Rh(CO)(Cl)(As((CH_2_)_14_)_3_As) (**14c**; 86%). The latter gave the iron tricarbonyl complex **7c** (51%), encountered in Scheme 2 as the precursor to **8c**. However, mainly starting materials were recovered after **8c** and Re(CO)_5_Cl were combined at 140 °C in chlorobenzene. In this case, the adduct *trans*-Re(CO)_3_(Cl)(As((CH_2_)_14_)_3_As) was sought as the diphosphine analog had been synthesized (via a metathesis/hydrogenation sequence) earlier.^35^

**Figure 5:**
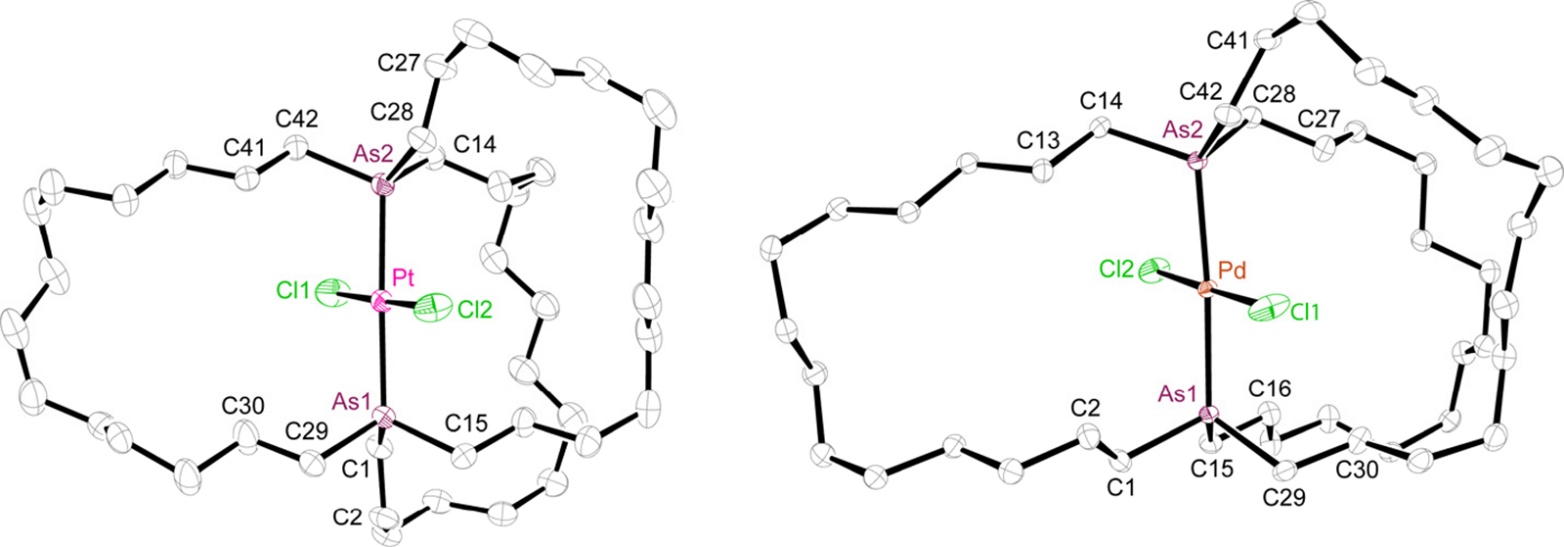
Thermal ellipsoid diagrams (50% probability) of platinum and palladium complexes **11c** and **12c** (dominant conformations only with hydrogen atoms omitted).

The crystal structures of the platinum and palladium complexes **11c** and **12c** could be determined. Molecular structures are given in Figure 5, and key distances and angles in Table S2. The former was isostructural with the diphosphorus analog *trans*-**3c** (Scheme 3).^11^ However, the latter differed from its congener, exhibiting four independent molecules in the unit cell. The arsenic–arsenic distances (4.7601(5)−4.8114(7) Å) were much shorter than those in *out,out*-**8c** (13.2873(15) Å), highlighting the conformational flexibility of the macrobicyclic ligand. As expected, they were slightly greater than the phosphorus–phosphorus distances in the corresponding diphosphine adducts (4.611–4.630 Å).^2a^

A final question concerns the thermodynamic partitioning of metal fragments between different macrobicycles E((CH_2_)_*n*_)_3_E as a function of the bridgehead atom or methylene chain length. Based upon relative donor strengths noted above,^8,19^ one would expect a diphosphine to bind preferentially to a diarsine. As shown in Scheme 7 (bottom), we were not able to establish equilibria. When an equimolar toluene-*d*_8_ solution of the iron diarsine complex **7c** and the diphosphine **1c** was kept at 100 °C for 24 h, no reaction was detected by ^31^P{^1^H} NMR. The sample was then irradiated at room temperature under the conditions used for **7c** in Scheme 5. Over the course of several h, **1c** was consumed but the characteristic signal of **5c** (71 ppm in toluene-*d*_8_) was not observed. Rather, three sharp signals at 62–66 ppm appeared as shown in Figure S4. These would be consistent a mixture of iron adducts, possibly oligomeric in nature.^36^

### Antimony and Bismuth Chemistry

**Scheme 8:**
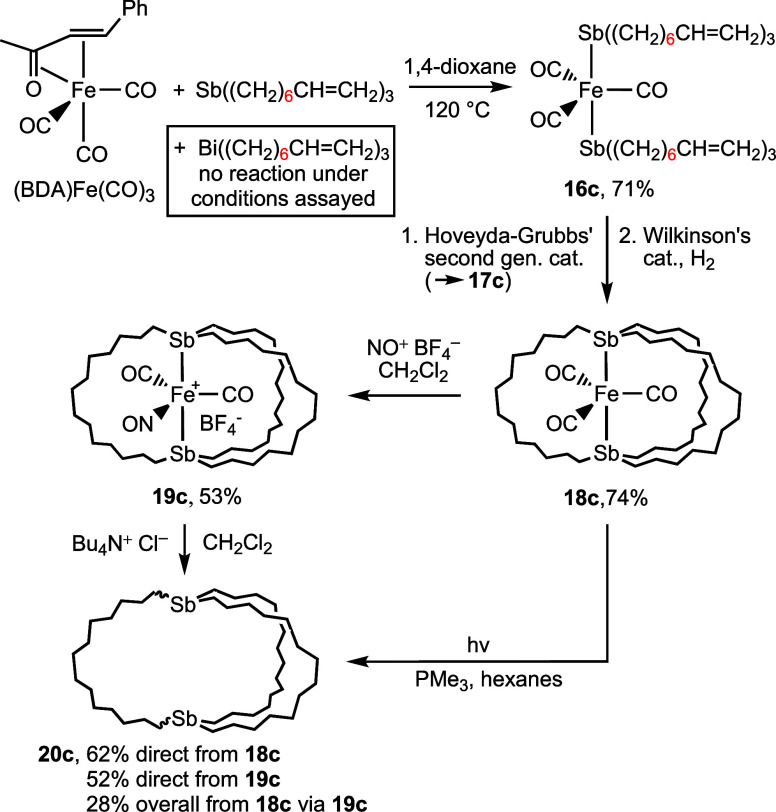
Syntheses of the Macrobicyclic Dibridgehead Distibine Sb((CH_2_)_14_)_3_Sb (**20c**)

As detailed in the introduction, antimony analogs of the preceding compounds were also sought, particularly due to the still greater length of the metal−pnictogen bond.^9^ Accordingly, the reaction of SbCl_3_ and the Grignard reagent BrMg(CH_2_)_6_CH=CH_2_ (3.01 equiv, THF) gave the trialkylstibine Sb((CH_2_)_6_CH=CH_2_)_3_ as an air stable colorless oil in 53% yield after workup. As shown in Scheme 8, a subsequent reaction with (BDA)Fe(CO)_3_ afforded the distibine complex *trans*-Fe(CO)_3_(Sb((CH_2_)_6_CH=CH_2_)_3_)_2_ (**16c**) as a dark red oil in 71% yield. However, this required much higher temperatures than with the triarsines As((CH_2_)_*m*_CH=CH_2_)_3_ in Scheme 5 (120 °C vs RT, 1,4-dioxane), indicating a greatly attenuated nucleophilicity and basicity.

In initial studies of **16c**, metathesis/hydrogenation sequences were carried out with Grubbs’ catalyst (18 mol % or 6 mol % per 2C=C) and Wilkinson’s catalyst (16 mol %), which contain PCy_3_ and PPh_3_ ligands. The results were disappointing, and some iron carbonyl phosphine adducts appeared to form. Thus, the phosphine-free Hoveyda−Grubbs’ second generation catalyst was employed, and the crude tris(olefin) *trans*-Fe(CO)_3_(Sb((CH_2_)_6_CH=CH(CH_2_)_6_)_3_Sb) (**17c**; mixture of *E*/*Z* isomers) was isolated by chromatography in 82% yield. This sample could be efficiently hydrogenated with Wilkinson’s catalyst, affording the target tricarbonyl complex *trans*-Fe(CO)_3_(Sb((CH_2_)_14_)_3_Sb) (**18c**) as a yellow powder in 90% yield, or 74% from **16c**.

Subsequent reaction of **18c** with NO^+^ BF_4_^–^ gave the nitrosyl adduct *trans*-[Fe(CO)_2_(NO)(Sb((CH_2_)_14_)_3_Sb)]^+^ BF_4_^–^ (**19c**, 53%). The IR ν_CO_ and ν_NO_ bands (2006/1949/1751 cm^–1^) were at lower frequencies than those of the diarsenic (**9c**; 2021/1961/1757 cm^–1^)^13^ and diphosphorus (2030/1965/1764 cm^–1^)^37^ analogs, suggesting that the metal fragment becomes *less* π basic as the pnictogen atom progresses from antimony to phosphorus. Although this runs counter to certain data involving the free ligands above,^19^ similar IR relationships are found for other series of triorganopnictogen carbonyl complexes.^9c,38^ This trend is less apparent with the tricarbonyl adducts **18c**, **7c**, and **5c** (Sb/As/P 1854/1855/1861 cm^–1^).

Paralleling Scheme 5, two approaches were taken to converting **18c** to the target dibridgehead distibine **20c**. First, photolysis of **18c** in the presence of PMe_3_ (10 equiv) afforded **20c** as a white solid in 62% yield. Second, treatment of **19c** with *n*-Bu_4_N^+^ Cl^–^ (1.6 equiv) gave **19c** in 52% yield, or 28% from **18c**. In contrast to the diarsine analogs, **20c** was quite air sensitive, with chromatographic purification best carried out in a glovebox.

The original goals of this project included the analogous dibridgehead dibisumuthanes Bi((CH_2_)_*n*_)_3_Bi and their metal complexes. Although Fe(CO)_4_ adducts of trialkylbismuth ligands have been isolated,^9c^ triphenylbismuth species have been problematic.^38^ The starting material Bi((CH_2_)_6_CH=CH_2_)_3_ could be synthesized analogously to the antimony homologue as described in the SI. However, subsequent treatment with (BDA)Fe(CO)_3_ did not give the desired product (toluene, 1,4-dioxane, and other solvents: 120 °C, 150 °C, >150 °C in sealed tubes, *h*ν).

## Discussion

This work has significantly advanced the synthesis and chemistry of macrocyclic dibridgehead di(trialkyl)pnictogens E((CH_2_)_*n*_)_3_E. Such dibridgehead diamines (Scheme 1) have been known since 1968,^1^ but except for two papers have not been further studied over the last 50 years.^39^ When their properties, the recently acquired data with the dibridgehead diphosphines **1a**–**e** (Schemes 2, 4)^2^ and the new results with the diarsines **8a**–**c** and distibine **20c** (Schemes 5–8) are interpreted together, many trends and relationships come into focus. Although the synthetic strategies used for **1**, **8**, and **20** do not appear extendable to analogous dibismuthanes, there is no reason to doubt their stabilities, and other pathways that do not require metal–bismuth intermediates may prove successful.^40^

These compounds can all be prepared in gram quantities,^2c^ so possible end-uses merit brief note. Organopnictogens are attracting attention in redox catalysis,^8^ and any mixed-valence radical anions derived from **1**, **8**, or **20** are certain to have fascinating properties and potential applications. Functional organoarsenic chemistry also continues to expand,^41^ now encompassing reagents in organic synthesis,^42^ designer ligands for metal catalysts,^43^ framework units in nanoporous materials,^44^ and biological probes.^45^ Organoantimony and bismuth compounds also play increasing roles in organic synthesis.^46^

**Scheme 9:**
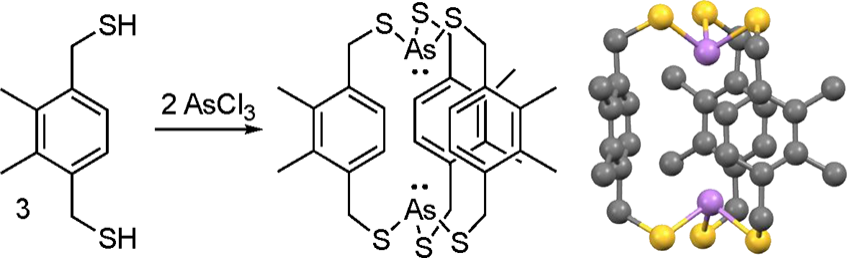
Macrobicyclic Dibridgehead Diarsenic Compounds with AsS_3_ Linkages

The chemistry most closely related to that in Scheme 5 has been developed by Johnson,^17^ who prepared a series of macrobicyclic dibridgehead diarsines that feature organosulfur linkers of the type SCH_2_(*p*-arylene)CH_2_S. An example is given in Scheme 9, and typical isolated yields are 31–54% These have only been accessed as *in*, *in* isomers, and the rigid arylene units should render homeomorphic isomerization challenging. Perhaps this could be facilitated by lengthening the CH_2_ segments, allowing access to *out*, *out* isomers. These compounds clearly have the potential for interesting coordination chemistry. Also, arsenic binding proteins feature cysteine derived As(SCH_2_R)_3_ centers that can have both *in* (*endo*) and *out* (*exo*) lone pair geometries.^47^

**Scheme 10:**
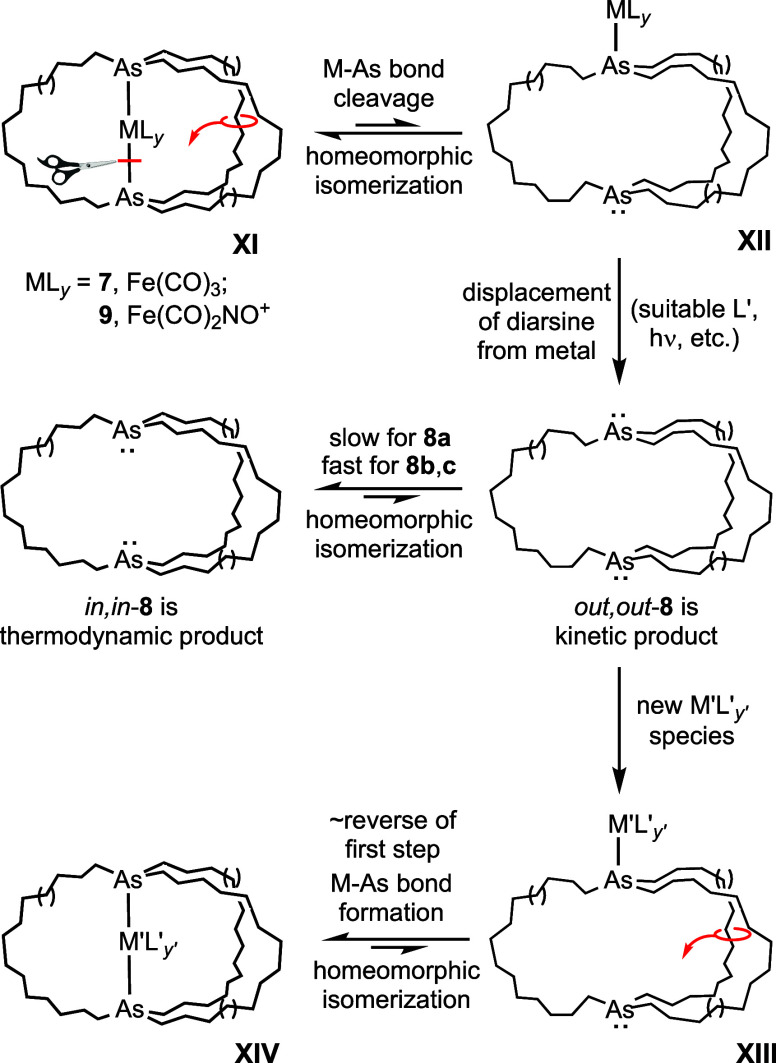
Possible Demetallation and “Remetallation” Mechanisms Involving Dibridgehead Diarsines

At first glance, the demetalation and/or “remetalation” reactions in Schemes 4, 5, 7, and 8 can seem puzzling, A proposal that mechanistically unifies these phenomena is presented in Scheme 10. We suggest that the means by which free diarsines are produced from the cationic nitrosyl complexes **9a**−**c** and *n*-Bu_4_N^+^ Cl^–^ involves (1) the cleavage of *one* iron–arsenic bond of **XI**, (2) homeomorphic isomerization to **XII**, and (3) displacement of the second arsenic atom by nucleophilic attack of the chloride anion at the metal. This initially affords *out,out*-**8**, which is the sole product detected in the case of **8a**, for which homeomorphic isomerization to *in,in*-**8a** is slow at room temperature.

It is well documented that in photolyses of iron carbonyl complexes, both carbonyl and ancillary ligands can be labilized.^48^ In theory, the same pathways should be available to **7**. Thus, diarsine photolabilization could be operative in the first step (**XI** → **XII**), the second (**XII** → *out,out*-**8**), or both. Evidence from another study^49^ suggests that irradiation can promote CO dissociation *directly* from the diphosphine tricarbonyl complexes **5a**−**e** (**XI**, ML_*y*_ = Fe(CO)_3_), without any accompanying homeomorphic isomerization. This might be the initial step in the conversion of **7a** to *in,in*-**8a**, which is the dominant photoproduct.

Most of the distinguishing structural features of the crystallographically characterized compounds have been highlighted above. One overarching property not treated is the degree of bridgehead pyramidalization, as reflected by the sums of the three CH_2_–As−CH_2_ bond angles (Table 1). Values for limiting geometries include 360° (trigonal planar), 328° (tetrahedral), and 270° (p_*x*_/p_*y*_/p_*z*_ array). The summed angles for *out,out*-**8c** (287.7°), *out,out*-**8a** (287.9°), and *out,out*-**8b** (287.9°, 288.3°) are not significantly different given the underlying esd values. Although *in,in*-**8a** (293.7°) is slightly more pyramidal, in all cases there is only a meager degree of s/p hybridization at arsenic.^7,8^ The CH_2_–P−CH_2_ angles in the phosphorus homologues *out,out*-**1a**,**b**,**c** sum to 295.2°, 295.4°/295.0°, and 295.9°, respectively, indicative of slightly less p character. The sum of the three CH_2_–As−CH_2_ angles in the diarsine dioxide **8c**·2O (331.4°, 328.1°) are in accord with a tetrahedral geometry, as seen for other arsine oxides.^30^

Looking to the future, it would be desirable to extend the quantitative rate and equilibrium data for the *in*/*out* isomers of the dibridgehead diphosphines **1** (Scheme 2) to the heavier pnictogens. However, since arsenic, antimony, and bismuth all lack nuclei with spin (*I*) = 1/2, practical NMR probes or “handles” are lacking. Accordingly, one approach will involve the dimethylation of the macrobicycles to the dications [H_3_CE((CH_2_)_*n*_)_3_ECH_3_]^2+^. This introduces two convenient nuclei for NMR studies. Although the rates and activation parameters will differ from those of the precursors, it will nevertheless help to define additional trends in rates of homeomorphic isomerization and attendant equilibria.

## Conclusion

To conclude, this study has established the ready availability of a family of structurally flexible macrocyclic dibridgehead di(trialkyl)arsines and di(trialkyl)stibines that represent attractive launching pads for a broad spectrum of subsequent chemistry, especially given their capability for *in*/*out* isomerism and homeomorphic isomerization. Their physical and chemical properties fill in many pieces of the partially completed jigsaw puzzle presented by the corresponding di(trialkyl)phosphines and di(trialkyl)amines at the outset of this study. And although a few pieces are still missing, the overall picture with respect to many properties and trends is now quite clear. Investigations of a variety of bridgehead derivatives are now in progress.

## Experimental Section

### General Methods

All reactions and workups were conducted under dry inert atmospheres in flame-dried glassware using conventional Schlenk techniques, except in the cases of As((CH_2_)_*n*_)_3_As (**8**), **8b**·2BH_3_, and **8c**·2O), for which workups were carried out in air. Sources of chemicals, instrumental methods, and related data are provided in the SI.

### As((CH_2_)_10_)_3_As (**8a**)

**A.** A Schlenk flask was charged with *trans*-[Fe(CO)_2_(NO)(As((CH_2_)_10_)_3_As)]^+^ BF_4_^–^ (**9a**; 0.158 g, 0.198 mmol),^13^*n*-Bu_4_N^+^ Cl^–^ (0.165 g, 0.594 mmol), and CH_2_Cl_2_ (15 mL) with stirring, and fitted with a condenser. The sample was refluxed (3 d). The solvent removed from the dark green solution by oil pump vacuum. The sample was dissolved in 1:1 v/v hexanes/CH_2_Cl_2_ and applied to a bed of neutral alumina (2.5 × 5.0 cm), which was washed with 1:1 v/v hexanes/CH_2_Cl_2_. The solvent was removed from the eluate by oil pump vacuum. The residue was rinsed with MeOH and EtOAc, collected by filtration, and dried by oil pump vacuum to give *out,out***-8a** as a white solid (0.038 g, 0.067 mmol, 34%), mp 112–114 °C (open capillary). Anal. Calcd for C_30_H_60_As_2_ (570.64): C, 63.14; H, 10.50. Found: C, 63.15; H, 10.61. **B.** A Schlenk flask was charged with *trans*-Fe(CO)_3_(As((CH_2_)_10_)_3_As) (**7a**; 0.040 g, 0.056 mmol),^13^ PMe_3_ (0.06 mL, 0.560 mmol), and hexanes (5 mL), and placed in front a water-cooled quartz immersion well of a Hanovia 450 W lamp (Figure S1). The sample was irradiated overnight with stirring. The solvent was removed by oil pump vacuum. The residue was dissolved in hexanes and applied to a short pipet column of silica gel. The column was rinsed with hexanes and the solvent was removed from the first fraction to give *in,in*-**8a** (0.013 g, 0.023 mmol, 41%) as a white solid, mp 53–57 °C (open capillary). Anal. Calcd for C_30_H_60_As_2_ (570.64): C, 63.14; H, 10.50. Found: C, 63.74; H, 10.80. Further elution gave *out,out***-8a**, which was similarly treated (0.004 g, 0.007 mmol, 13%).

NMR (*out,out*-**8a**, CDCl_3_, δ/ppm): ^1^H (500 MHz) 1.50–1.43 (m, 12H), 1.41–1.28 (m, 48H); ^13^C{^1^H} (125 MHz)^21^ 31.0 (s, As**C**H_2_), 27.8 (s, AsCH_2_CH_2_CH_2_CH_2_**C**H_2_), 26.9 (s, AsCH_2_CH_2_CH_2_**C**H_2_), 25.3 (s, AsCH_2_CH_2_**C**H_2_), 23.3 (s, AsCH_2_**C**H_2_).

NMR (*in,in*-**8a**, CDCl_3_, δ/ppm): ^1^H (500 MHz) 1.56–1.50 (m, 12H), 1.46–1.48 (m, 24H), 1.37–1.29 (m, 24H); ^13^C{^1^H} (125 MHz) 29.9 (s, As**C**H_2_), 28.0 (s, AsCH_2_CH_2_CH_2_CH_2_**C**H_2_), 27.5 (s, AsCH_2_CH_2_CH_2_**C**H_2_), 26.9 (s, AsCH_2_CH_2_**C**H_2_), 26.7 (s, AsCH_2_**C**H_2_). HRMS (APCI, *m*/*z*): calcd for C_30_H_60_As_2_ [M + H]^+^, 571.3200. Found, 571.3194.

### As((CH_2_)_12_)_3_As (**8b**)

**A.** A Schlenk flask was charged with *trans*-[Fe(CO)_2_(NO)(As((CH_2_)_12_)_3_As)]^+^ BF_4_^–^ (**9b**; 0.050 g, 0.056 mmol),^13^*n*-Bu_4_N^+^ Cl^–^ (0.023 g, 0.085 mmol), and CH_2_Cl_2_ (8 mL) with stirring. After 14 h, the solvent was removed from the dark green solution by oil pump vacuum. The sample was dissolved in 1:1 v/v hexanes/CH_2_Cl_2_ and applied to a bed of neutral alumina (2.5 × 5.0 cm), which was washed with 1:1 v/v hexanes/CH_2_Cl_2_. The solvent was removed from the eluate by oil pump vacuum to give **8b** as a white solid (0.028 g, 0.043 mmol, 77%), mp 54–56 °C (open capillary). Anal. Calcd for C_36_H_72_As_2_ (654.80): C, 66.03; H, 11.08. Found: C, 65.94; H, 11.10. **B.** A Schlenk flask was charged with *trans*-Fe(CO)_3_(As((CH_2_)_12_)_3_As) (**7b**; 0.151 g, 0.190 mmol),^13^ PMe_3_ (0.20 mL, 1.900 mmol), and hexanes (10 mL), and placed in front of a water-cooled quartz immersion well of a Hanovia 450 W lamp (Figure S1). The sample was irradiated overnight with stirring. The solvent was removed by oil pump vacuum. The residue was dissolved in hexanes and applied to a short pipet column of silica gel. The column was rinsed with hexanes and the solvent was removed from the eluate by oil pump vacuum to afford **8b** (0.086 g, 0.131 mmol, 69%) as a white solid.

NMR (toluene-*d*_8_, δ/ppm): ^1^H (500 MHz) 1.60–1.50 (m, 12H), 1.47–1.38 (m, 22H), 1.37–1.26 (m, 38H); ^13^C{^1^H} (125 MHz; see also additional data in Figure S2)^21^ 31.4 (s, As**C**H_2_), 28.7 (s, AsCH_2_CH_2_CH_2_CH_2_CH_2_**C**H_2_), 28.6 (s, AsCH_2_CH_2_CH_2_CH_2_**C**H_2_), 28.5 (s, AsCH_2_CH_2_CH_2_**C**H_2_), 26.6 (s, AsCH_2_CH_2_**C**H_2_), 25.3 (br s, AsCH_2_**C**H_2_).

### As((CH_2_)_14_)_3_As (**8c**)

**A.***trans*-[Fe(CO)_2_(NO)(As((CH_2_)_14_)_3_As)]^+^ BF_4_^–^ (**9c**; 0.170 g, 0.175 mmol),^13^*n*-Bu_4_N^+^ Cl^–^ (0.073 g, 0.26 mmol), and CH_2_Cl_2_ (26 mL) were combined in a procedure analogous to that for **8b**. An identical workup gave **8c** as a white solid (0.097 g, 0.131 mmol, 75%), mp 62–64 °C (open capillary). Anal. Calcd for C_42_H_84_As_2_ (738.96): C, 68.26; H, 11.46. Found: C, 68.08; H, 11.43. **B.***trans*-Fe(CO)_3_(As((CH_2_)_14_)_3_As) (**7c**; 0.080 g, 0.091 mmol),^13^ PMe_3_ (0.09 mL, 0.910 mmol), and hexanes (4 mL) were combined in a procedure analogous to that for **8b**. An identical workup gave **8c** (0.053 g, 0.072 mmol, 79%) as a white solid. Anal. Calcd for C_42_H_84_As_2_ (738.96): C, 68.26; H, 11.46. Found: C, 68.05; H, 11.51.

NMR (toluene-*d*_8_, δ/ppm): ^1^H (500 MHz) 1.55–1.45 (m, 12H), 1.41–1.34 (m, 22H), 1.33–1.22 (m, 50H); ^13^C{^1^H} (125 MHz; see also additional data in Figure S3)^21^ 32.0 (s, As**C**H_2_), 29.7 (s, AsCH_2_CH_2_CH_2_CH_2_CH_2_CH_2_**C**H_2_), 29.61 (s, AsCH_2_CH_2_CH_2_CH_2_CH_2_**C**H_2_), 29.57 (s, AsCH_2_CH_2_CH_2_CH_2_**C**H_2_), 29.2 (s, AsCH_2_CH_2_CH_2_**C**H_2_), 27.0 (s, AsCH_2_CH_2_**C**H_2_), 25.2 (s, AsCH_2_**C**H_2_). HRMS (APCI, *m*/*z*): calcd for C_42_H_84_As_2_ [M + H]^+^, 739.5078. Found, 739.5116.

### As((CH_2_)_12_)_3_As·2BH_3_ (**8b**·2BH_3_)

A Schlenk flask was charged with **8b** (0.142 g, 0.217 mmol), Me_2_S·BH_3_ (2.0 M in THF; 0.73 mL, 1.457 mmol), and THF (4 mL) with stirring. After 3 d, the solvent was removed from the clear solution by oil pump vacuum. The sample was dissolved in hexanes and applied to a bed of silica gel (2.5 × 5.0 cm), which was washed with hexanes and then CH_2_Cl_2_. The solvent was removed from the eluate by oil pump vacuum to give **8b**·2BH_3_ as a clear oil (0.139 g, 0.204 mmol, 94%). Anal. Calcd for C_36_H_78_As_2_B_2_ (682.47): C, 63.36; H, 11.52. Found: C, 63.42; H, 11.41.

NMR (CDCl_3_, δ/ppm): ^1^H (500 MHz) 1.68–1.61 (br m, 12H), 1.57–1.47 (m, 12H), 1.42–1.35 (br m, 12H), 1.34–1.26 (br m, 36H), 0.88–1.56 (br s, 6H, BH_3_); ^13^C{^1^H} (125 MHz) 30.7 (s, As**C**H_2_), 28.9 (s, AsCH_2_CH_2_CH_2_CH_2_CH_2_**C**H_2_), 28.6 (s, AsCH_2_CH_2_CH_2_CH_2_**C**H_2_), 28.1 (s, AsCH_2_CH_2_CH_2_**C**H_2_), 23.3 (s, AsCH_2_CH_2_**C**H_2_), 21.2 (s, AsCH_2_**C**H_2_).

### O=As((CH_2_)_14_)_3_As=O (**8c**·2O)

A vial was charged with **8c** (0.083 g, 0.112 mmol) and CH_2_Cl_2_ (2 mL). Then H_2_O_2_ (35 wt %, 2.0 mL, 20.58 mmol) was slowly added with stirring. After 14 h, methanol and CH_2_Cl_2_ were added and the solvent was removed in vacuo. Then 3 Å molecular sieves and toluene (20 mL) was added. The sample was stirred (14 h) and filtered. The filter pad was rinsed with toluene and CH_2_Cl_2_. The solvent was removed from the filtrate in vacuo and the residue triturated with hexanes. The sample was dried in vacuo to give **8c**·2O as a white solid.^28^ (0.073 g, 0.095 mmol, 85%), mp 88–90 °C (open capillary). Anal. Calcd for C_42_H_84_As_2_O_2_·(H_2_O)_4_ (842.03): C, 59.84; H, 11.00. Found: C, 60.13; H, 10.71. Hydrated samples may be dried with 3 Å molecular sieves.

NMR (CDCl_3_, δ/ppm): ^1^H (500 MHz) 1.91–1.79 (br m, 12H), 1.63–1.49 (12H, br m), 1.40–1.29 (12H, br m), 1.27–1.15 (br m, 48H); ^13^C{^1^H} (125 MHz)^28^ 30.8 (s, CH_2_), 30.7 (s, CH_2_), 29.2 (br s, CH_2_), 29.0 (s, CH_2_), 28.9 (s, CH_2_), 28.8 (s, CH_2_), 28.7 (s, CH_2_), 28.5 (s, CH_2_), 28.4 (s, CH_2_), 22.3 (s, CH_2_), 22.2 (s, CH_2_). IR (powder film, cm^–1^): 2914 (s), 2849 (s), 1742 (w), 1462 (m), 1373 (w), 1261 (w), 1175 (w), 1097 (m), 1022 (w), 881 (s, ν_As=O_),^26^ 808 (m), 717 (m), 667 (w). HRMS (APCI, *m*/*z*): calcd for C_42_H_84_As_2_O_2_ [M + H]^+^, 771.4976. Found, 771.4952.

### *trans*-Pt(Cl)_2_(As((CH_2_)_14_)_3_As) (**11c**)

A Schlenk flask was charged with **8c** (0.100 g, 0.135 mmol), PtCl_2_ (0.044 g, 0.169 mmol), and toluene (8 mL) with stirring. After 18 h, the solvent was removed by oil pump vacuum. The residue was dissolved in 1:1 v/v hexanes/CH_2_Cl_2_ and applied to a bed of neutral alumina (2.5 × 5.0 cm), which was eluted with 1:1 v/v hexanes/CH_2_Cl_2_. The solvent was removed from the eluate by oil pump vacuum to give a yellow oil, to which 1:1 v/v EtOAc/MeOH was added. The solvents were removed by oil pump vacuum to give **11c** as a pale yellow solid (0.117 g, 0.116 mmol, 86%), mp 117–119 °C (open capillary). Anal. Calcd for C_42_H_84_As_2_Cl_2_Pt (1104.95): C, 50.20; H, 8.43. Found: C, 50.69; H, 8.69.

NMR (CDCl_3_, δ/ppm): ^1^H (500 MHz) 1.89–1.83 (br m, 12H), 1.78–1.70 (m, 12H), 1.48–1.42 (br m, 12H), 1.37–1.29 (br m, 48H); ^13^C{^1^H} (125 MHz)^34^ 30.7 (s, AsCH_2_CH_2_**C**H_2_), 28.2 (s, As**C**H_2_), 27.5 (s, CH_2_), 27.44 (s, CH_2_), 27.40 (s, CH_2_), 24.6 (s, CH_2_), 21.3 (s, AsCH_2_**C**H_2_).

### *trans*-Pd(Cl)_2_(As((CH_2_)_14_)_3_As) (**12c**)

A Schlenk flask was charged with **8c** (0.100 g, 0.135 mmol), PdCl_2_ (0.030 g, 0.169 mmol), and CH_2_Cl_2_ (6 mL) with stirring. After 4 d, the solvent was removed by oil pump vacuum. The residue was dissolved in CH_2_Cl_2_ and applied to a bed of Celite (2.5 × 5.0 cm), which was rinsed with CH_2_Cl_2_. The solvent was removed from the eluate by oil pump vacuum to give a waxy yellow solid, and 1:1 v/v EtOAc/MeOH was added. The solvent was removed by oil pump vacuum to give **12c** as a pale yellow solid (0.097 g, 0.105 mmol, 78%), dec pt. 193 °C (open capillary). Anal. Calcd for C_42_H_84_As_2_Cl_2_Pd (916.29): C, 55.05; H, 9.24. Found: C, 55.19; H, 9.14.

NMR (CDCl_3_, δ/ppm): ^1^H (500 MHz) 1.91–1.81 (br m, 12H), 1.77–1.66 (m, 12H), 1.48–1.40 (br m, 12H), 1.37–1.26 (br m, 48H); ^13^C{^1^H} (125 MHz)^34^ 30.9 (s, AsCH_2_CH_2_**C**H_2_), 28.2 (s, As**C**H_2_), 27.6 (s, CH_2_), 27.44 (s, CH_2_), 27.42 (s, CH_2_), 25.0 (s, CH_2_), 22.7 (s, AsCH_2_**C**H_2_).

### *trans*-Ni(Cl)_2_(As((CH_2_)_14_)_3_As) (**13c**) A

A Schlenk flask was charged with **8c** (0.033 g, 0.045 mmol), NiCl_2_(NCCH_3_) (0.012 g, 0.056 mmol),^50^ and THF (5 mL) with stirring. After 18 h, the solvent was removed by oil pump vacuum. Then CH_2_Cl_2_ was added and the sample filtered through a glass frit. The solvent was removed from the filtrate by oil pump vacuum and the residue triturated with EtOAc. The sample was dried by oil pump vacuum to give **13c** as a purple solid (0.031 g, 0.036 mmol, 80%). **B.** A Schlenk flask was charged with **8c** (0.100 g, 0.135 mmol), NiCl_2_(DME) (0.037 g, 0.169 mmol), and THF (15 mL) with stirring. After 18 h, the solvent was removed by oil pump vacuum. Then CH_2_Cl_2_ was added and the sample filtered through a glass frit. The solvent was removed from the filtrate by oil pump vacuum, and the solid rinsed with EtOAc into a filter frit. The filter cake was eluted with CH_2_Cl_2_, and the CH_2_Cl_2_ removed by oil pump vacuum to give **13c** as a purple solid (0.080 g, 0.092 mmol, 68%), dec pt. 206 °C (open capillary). Anal. Calcd for C_42_H_84_As_2_Cl_2_Ni (868.56): C, 58.08; H, 9.75. Found: C, 58.33; H, 9.90.

NMR (CDCl_3_, δ/ppm): ^1^H (500 MHz) 1.95–1.81 (br m, 12H), 1.76–1.66 (br m, 12H), 1.56–1.47 (br m, 12H), 1.43–1.22 (br m, 48H); ^13^C{^1^H} (125 MHz)^34^ 31.1 (s, AsCH_2_CH_2_**C**H_2_), 28.2 (s, As**C**H_2_), 27.6 (s, CH_2_), 27.4 (s, CH_2_), 25.1 (s, CH_2_), 22.1 (s, AsCH_2_**C**H_2_).

### *trans*-Rh(CO)(Cl)(As((CH_2_)_14_)_3_As) (**14c**)

A Schlenk flask was charged with **8c** (0.147 g, 0.199 mmol), [Rh(Cl)(COD)]_2_ (0.059 g, 0.119 mmol), and 1:1 v/v hexanes/CH_2_Cl_2_ (30 mL). Then CO was aspirated through the solution. The sample was stirred under a closed CO atmosphere (18 h), and the solvent was removed by oil pump vacuum. The residue was dissolved in 1:1 v/v hexanes/CH_2_Cl_2_ and applied to a bed of neutral alumina (2.5 × 5.0 cm), which was washed with 1:1 v/v hexanes/CH_2_Cl_2_. The solvent was removed from the eluate by oil pump vacuum. The residue was triturated several times with EtOAc and dried in vacuo to give **14c** as a pale yellow solid (0.155 g, 0.171 mmol, 86%), dec pt. at 146 °C (open capillary). Anal. Calcd for C_43_H_84_As_2_ClORh (905.33): C, 57.05; H, 9.35. Found: C, 56.98; H, 9.44.

NMR (CDCl_3_, δ/ppm): ^1^H (500 MHz) 1.91–1.81 (br m, 12H), 1.72–1.63 (m, 12H), 1.45–1.39 (br m, 12H), 1.35–1.27 (br m, 48H); ^13^C{^1^H} (125 MHz)^34^ 186.9 (s, CO), 31.0 (s, AsCH_2_CH_2_**C**H_2_), 28.1 (s, As**C**H_2_), 27.9 (s, CH_2_), 27.5 (s, CH_2_), 27.4 (s, CH_2_), 25.6 (s, CH_2_), 24.9 (s, AsCH_2_**C**H_2_). IR (powder film, cm^–1^): 2918 (w), 2849 (w), 1942 (s, ν_C≡O_), 1457 (m), 1260 (w), 1093 (w), 1017 (m), 801 (m), 719 (m).

### *trans*-Fe(CO)_3_(As((CH_2_)_14_)_3_As) (**7c**)

A Schlenk flask was charged with **8c** (0.029 g, 0.039 mmol), (BDA)Fe(CO)_3_ (0.014 g, 0.049 mmol),^51^ and THF (5 mL) with stirring. After 18 h, the solvent was removed by oil pump vacuum. The sample was dissolved in 2:1 v/v hexanes/CH_2_Cl_2_ and applied to a bed of neutral alumina (2.5 × 5.0 cm), which was washed with 2:1 v/v hexanes/CH_2_Cl_2_. The solvent was removed from the eluate by oil pump vacuum to give **8c**(13) as a white-yellow solid (0.018 g, 0.020 mmol, 51%).

### *trans*-Fe(CO)_3_(Sb((CH_2_)_6_CH=CH_2_)_3_)_2_ (**16c**)

A Schlenk flask was charged with (BDA)Fe(CO)_3_ (0.459 g, 1.61 mmol),^51^ 1,4-dioxane (30 mL), and Sb((CH_2_)_6_CH=CH_2_)_3_ (1.54 g, 3.37 mmol; see SI) with stirring and fitted with a condenser. The mixture was kept at 120 °C. After 16 h, the solvent was removed by oil pump vacuum. The residue was dissolved in hexanes and applied to a short pad of silica (2.5 × 5.0 cm), which was washed with hexanes. The product was then eluted with CH_2_Cl_2_. The solvent was removed from the eluate by oil pump vacuum to give **16c** as a dark red oil (1.21 g, 1.15 mmol, 71%). Anal. Calcd for C_51_H_90_FeO_3_Sb_2_ (1050.64): C 58.30: H 8.63. Found: C 58.17; H 8.58.

NMR (CDCl_3_, δ/ppm): ^1^H (500 MHz) 5.81 (ddt, ^3^*J*_HH*trans*_ = 16.9 Hz, ^3^*J*_HH*cis*_ = 10.2 Hz, ^3^*J*_HH_ = 6.7 Hz, 6H), 5.05–4.86 (m, 12H), 2.07–2.01 (m, 12H), 1.85–1.75 (m, 12H), 1.69–1.62 (m, 12H), 1.42–1.33 (m, 36H); ^13^C{^1^H} (125 MHz) 213.9 (s, CO), 139.1 (s, **C**H = ), 114.2 (s, = **C**H_2_), 33.8 (s, **C**H_2_CH=CH_2_), 32.6 (s, SbCH_2_CH_2_**C**H_2_), 28.8 (s, CH_2_), 28.6 (s, CH_2_), 25.7 (s, Sb**C**H_2_), 15.8 (s, SbCH_2_**C**H_2_). IR (oil film, cm^–1^): 2924 (m), 2854 (m), 1854 (s, ν_C≡O_), 1640 (w), 1456 (w), 991 (m), 907 (s), 687 (w), 629 (s).

### *trans*-Fe(CO)_3_(Sb((CH_2_)_6_CH=CH((CH_2_)_6_)_3_Sb) (**17c**)

A 3-neck round-bottom flask was charged with **16c** (1.322 g, 1.26 mmol) and CH_2_Cl_2_ (700 mL; the resulting solution is 0.0018 M in **16c**) and fitted with a condenser. A solution of Hoveyda−Grubbs’ second generation catalyst was prepared (0.142 g (0.226 mmol, 18 mol %) in 10 mL of CH_2_Cl_2_). Half was added dropwise over 5 min. The sample was refluxed. After 18 h, the remaining catalyst was added. After another 16 h, the solvent was removed by oil pump vacuum. The residue was filtered through neutral alumina (2.5 × 5 cm) using 2:1 v/v hexanes/CH_2_Cl_2_. The solvent was removed from the filtrate by oil pump vacuum to give **17c** as a yellow-orange powder (0.993 g, 1.027 mmol, 82%, mixture of *E*/*Z* isomers), dec pt. 120 °C (open capillary). A smaller scale reaction is described in the SI.

NMR (CDCl_3_, δ/ppm): ^1^H (500 MHz) 5.44–5.25 (m, 6H), 2.13–1.93 (m, 12H), 1.89–1.58 (m, 24H), 1.45–1.28 (m, 36H). ^13^C{^1^H} (125 MHz; major isomer only) 213.6 (s, CO), 131.0 (s, **C**H=), 32.8 (s, **C**H_2_CH=CH_2_), 32.5 (s, SbCH_2_CH_2_**C**H_2_), 28.8 (s, CH_2_), 28.4 (s, CH_2_), 26.3 (s, CH_2_), 16.6 (s, SbCH_2_**C**H_2_). IR (powder film, cm^–1^): 2920 (s), 2849 (m), 2038 (m), 1928 (m), 1853 (s, ν_C≡O_), 1462 (m), 1260 (m), 1018 (m), 966 (m), 799 (m), 715 (w), 686 (m), 630 (s).

### *trans*-Fe(CO)_3_(Sb((CH_2_)_14_)_3_Sb) (**18c**)

A Fischer−Porter bottle was charged with **17c** (0.993 g, 1.027 mmol), ClRh(PPh_3_)_3_ (0.156 g, 0.164 mmol, 16 mol %), toluene (30 mL), and H_2_ (85 psig). The solution was stirred at 80 °C. After 72 h, the solvent was removed by oil pump vacuum. The residue was filtered through neutral alumina (2.5 × 5.0 cm) using 2:1 v/v hexane/CH_2_Cl_2_. The solvent was removed from the filtrate by oil pump vacuum to give **18c** as a yellow powder (0.899 g, 0.924 mmol, 90%), dec pt. 130 °C (open capillary). Anal. Calcd for C_45_H_84_FeO_3_Sb_2_ (972.53): C, 55.58; H, 8.71. Found: C, 56.08; H, 8.55.

NMR (CDCl_3_, δ/ppm): ^1^H (500 MHz) 1.84–1.75 (m, 12H), 1.74–1.66 (m, 12H), 1.45–1.36 (m, 12H), 1.35–1.25 (m, 48H); ^13^C{^1^H} (125 MHz) 214.0 (s, CO), 32.2 (s, SbCH_2_CH_2_**C**H_2_), 28.1 (s, Sb**C**H_2_), 28.0 (s, CH_2_), 27.3 (s, CH_2_), 27.1 (s, CH_2_), 25.5 (s, CH_2_), 16.8 (s, SbCH_2_**C**H_2_). IR (powder film, cm^–1^): 2921 (s), 2852 (m), 1854 (s, ν_C≡O_), 1456 (w), 742 (w), 693 (m), 629 (s).

### *trans*-[Fe(CO)_2_(NO)(Sb((CH_2_)_14_)_3_Sb)]^+^ BF_4_^–^ (**19c**)

A Schlenk flask was charged with **18c** (0.072 g, 0.074 mmol) and CH_2_Cl_2_ (4 mL). Solid NO^+^ BF_4_^–^ (0.013 g, 0.111 mmol) was added with stirring. After 72 h, the solvent was removed from the pale yellow mixture by oil pump vacuum, and CH_2_Cl_2_ added. The mixture was filtered through a short pipet column of Celite, which was washed with CH_2_Cl_2_. The solvent was removed from the filtrate by oil pump vacuum to give a waxy residue. The residue was triturated with hexanes and diethyl ether, and dissolved in 1:1 hexanes/MeOH. The sample was taken to dryness by oil pump vacuum to give **19c** as a yellow solid (0.042 g, 0.039 mmol, 53%), dec pt. 191 °C (open capillary). Anal. Calcd for C_44_H_84_BF_4_FeNO_3_Sb_2_ (1061.33): C, 49.79; H, 7.98. Found: C, 50.56; H, 7.81.^52^

NMR (CDCl_3_, δ/ppm): ^1^H (500 MHz) 2.64–2.34 (m, 12H), 1.81–1.61 (m, 12H), 1.51–1.15 (m, 60H); ^13^C{^1^H} (125 MHz) 207.4 (s, CO), 31.8 (s, SbCH_2_CH_2_**C**H_2_), 27.8 (s, Sb**C**H_2_), 27.6 (s, CH_2_), 27.1 (s, CH_2_), 26.6 (s, CH_2_), 26.1 (s, CH_2_), 17.8 (s, SbCH_2_**C**H_2_). IR (powder film, cm^–1^): 2922 (m), 2851 (m), 2006 (w, ν_C≡O_), 1949 (s, ν_C≡O_), 1751 (s, ν_N≡O_), 1457 (w), 1056 (s), 696 (w), 623 (s).

### Sb((CH_2_)_14_)_3_Sb (**20c**)

**A.** A Schlenk flask was charged with **19c** (0.143 g, 0.135 mmol), *n*-Bu_4_N^+^ Cl^–^ (0.056 g, 0.202 mmol), and CH_2_Cl_2_ (10 mL) with stirring. After 14 h, the solvent was removed from the dark brown solution by oil pump vacuum. The sample was dissolved in hexanes and applied to a pipet column of silica gel. The column was rinsed with hexanes and the solvent removed from the filtrate by oil pump vacuum to give **20c** (0.059 g, 0.071 mmol, 52%), dec pt. 258 °C (closed capillary). Anal. Calcd for C_42_H_84_Sb_2_ (832.65): C, 60.58; H, 10.17. Found: C, 60.51; H, 10.18. **B. 18c** (0.084 g, 0.086 mmol), PMe_3_ (0.090 mL, 0.86 mmol), and hexanes (6 mL) were combined in a photochemical procedure analogous to that for **8b**. An identical workup gave **20c** (0.044 g, 0.053 mmol, 62%) as a white solid.

NMR (CDCl_3_, δ/ppm): ^1^H (500 MHz) 1.59–1.48 (m, 12H), 1.41–1.25 (m, 72H); ^13^C{^1^H} (125 MHz)^21^ 33.3 (s, Sb**C**H_2_), 29.5 (s, SbCH_2_CH_2_CH_2_CH_2_CH_2_CH_2_**C**H_2_), 29.39 (s, SbCH_2_CH_2_CH_2_CH_2_CH_2_**C**H_2_), 29.37 (s, SbCH_2_CH_2_CH_2_CH_2_**C**H_2_), 28.7 (SbCH_2_CH_2_CH_2_**C**H_2_CH_2_), 27.5 (s, SbCH_2_CH_2_**C**H_2_), 17.8 (s, SbCH_2_**C**H_2_).

## Supporting Information Available

Additional syntheses, and crystallographic and characterization data (^1^H/^13^C{^1^H}/^31^P{^1^H} NMR, IR); photolysis apparatus; variable temperature NMR and reaction monitoring by NMR (PDF)
