# Supplementary material for: Macrobicyclic Dibridgehead Di(trialkyl)pnictogens E((CH2)n)3E (E/n = As/10, As/12, As/14, Sb/14) and Their Cage-like Metal Complexes: Syntheses, Structures, and Homeomorphic Isomerizations
Source: Organometallics. 2024 May 21;43(11):1285–98. doi: 10.1021/acs.organomet.4c00120 (PMC11449212; doi:10.1021/acs.organomet.4c00120)
Supplement: Supplementary file 1 — om4c00120_si_001.pdf [file om4c00120_si_001.pdf]

SUPPORTING INFORMATION FOR

Macrobicyclic Dibridgehead Di(trialkyl)pnictogens $E((CH_2)_n)_3E$ (E/n = As/10, As/12, As/14, Sb/14) and their Cage-Like Metal Complexes: Syntheses, Structures, and Homeomorphic Isomerizations

Samuel R. Zarcone, Peter J. Verardi, Gong M. Chu, Nattamai Bhuvanesh, and John A. Gladysz*

Department of Chemistry, Texas A&M University, PO Box 30012, College Station, Texas 77842-3012, USA E-mail: gladysz@mail.chem.tamu.edu

submitted to *Organometallics*

■ EXPERIMENTAL SECTION

General. Reactions were conducted under nitrogen atmospheres unless otherwise noted. Materials were utilized as follows: CH_2Cl_2, THF, hexanes, and toluene, purified by a Glass Contour system; EtOAc, 1,4-dioxane, MeOH (3 × ≥99.5%, Sigma Aldrich), $CDCl_3$, CD_2Cl_2, toluene-d_8, C_6D_5Br (4 × Cambridge Isotopes), Mg turnings (Alfa Aesar), $Br(CH_2)_3CH{=}CH_2$ (99%, TCI), $Br(CH_2)_6CH{=}CH_2$ (98%, aablocks), 1,2-dibromoethane (99%, Sigma Aldrich), n-Bu_4N^+ Cl^- (≥97%, Sigma Aldrich), NO^+ BF_4^- (Alfa Aesar, 98%), PMe_3 (98%, Strem), H_2O_2 (35 wt%, BDH), $Me_2S{\cdot}BH_3$ (2.0 M in THF, Sigma Aldrich), $PtCl_2$ (98%, Ambeed), $PdCl_2$ (60% Pd, Pressure Chemicals), $NiCl_2$(DME) (98%, Ambeed), $[Rh(Cl)(COD)]_2$ (42% Rh, Pressure Chemicals), $AsCl_3$ (≥99%, Sigma Aldrich), $SbCl_3$ (99%, Alfa Aesar), $BiCl_3$ (97%, TCI), Hoveyda-Grubbs' 2nd generation catalyst (98%, A2B Chem), $ClRh(PPh_3)_3$ (99%, Sigma-Aldrich), Celite (EMD), silica gel (40-63 μm mesh, Silicycle), and neutral alumina (Brockmann I, for chromatography, 40-300 μm mesh, 60A, Acros), used as received. Molecular sieves (3Å, Beantown Chemical) were heated under vacuum (120 °C, 12 h).

NMR spectra were recorded on standard FT spectrometers at ambient probe temperatures unless noted and referenced as follows (δ/ppm): ^1H: residual internal $CHCl_3$ (7.26), $C_6D_5CHD_2$ (2.08); ^{13}C {^1H}: internal $CDCl_3$ (77.16), $C_6D_5\underline{\mathbf{C}}D_3$ (20.43). IR spectra were recorded using a Shimadzu IRAffinity-1 spectrometer with a Pike MIRacle ATR system (diamond/ ZnSe crystal). Atmospheric pressure chemical ionization mass spectra (APCI-MS) were obtained with a Thermo Scientific Q Exactive Focus instrument. Melting points were determined on a Stanford Research Systems (SRS) MPA100 (OptiMelt) automated device. Microanalyses were conducted by Atlantic Microlab.

Syntheses of New Trialkylpnictogens and Homologs of Previously Reported Compounds

$As((CH_2)_3CH{=}CH_2)_3$. A flame dried Schlenk flask was charged with magnesium turnings (1.92 g, 78.8 mmol), THF (40 mL), and 1,2-dibromoethane (0.40 mL, 4.6 mmol), and cooled to 0 °C. Then $Br(CH_2)_3CH{=}CH_2$ (3.90 g, 3.10 mL, 26.2 mmol) was added dropwise with stirring over 10 min. After 1 h, the cold bath was removed. After 16 h, the remaining magnesium was removed

by filtration. Following a standard protocol,[s1] an aliquot of the filtrate was used to determine the concentration of the Grignard reagent $BrMg(CH_2)_3CH{=}CH_2$. The filtrate was cooled to 0 °C, and $AsCl_3$ (1.23 g, 0.57 mL, 6.81 mmol; 1:3 As/RMgBr) in THF (5 mL) was added dropwise with stirring over 10 min. The cold bath was removed. After 16 h, the mixture was cooled to 0 °C and aqueous NH_4Cl (20 mL) was added dropwise with stirring over 10 min. After 1 h, the cold bath was removed. After 1 h, the aqueous phase was removed via syringe. The solvent was removed from the organic phase by oil pump vacuum, and CH_2Cl_2 (20 mL) was added. The solution was passed through a short pad of silica (2.5 × 5.0 cm), which was washed with CH_2Cl_2. The solvent was removed from the filtrate by oil pump vacuum to give $As((CH_2)_3CH{=}CH_2)_3$ as a colorless oil (1.80 g, 6.39 mmol, 94%). Anal. Calcd. For $C_{15}H_{27}As$ (282.30): C, 63.82; H, 9.64. Found: C, 64.03; H, 9.54.

NMR ($CDCl_3$, δ/ppm): ^1H (500 MHz) 5.79 (ddt, $^3J_{HHtrans}$ = 17.0 Hz, $^3J_{HHcis}$ = 10.2 Hz, $^3J_{HH}$ = 6.7 Hz, 3H), 5.16-4.88 (m, 6H), 2.15-2.07 (m, 6H), 1.59-1.51 (m, 6H), 1.44-1.39, (m, 6H); $^{13}C\{^1H\}$ (125 MHz) 138.6 (s, **C**H=), 114.9 (s, =**C**H_2), 36.1 (s, $AsCH_2CH_2$**C**H_2), 26.4 (s, As**C**H_2), 24.7 (s, $AsCH_2$**C**H_2).

***trans*-Fe(CO)$_3$(As((CH$_2$)$_3$CH=CH$_2$)$_3$)$_2$.** A flame dried Schlenk flask was charged with (BDA)$Fe(CO)_3$ (0.868 g, 3.03 mmol),[s2] THF (20 mL), and $As((CH_2)_3CH{=}CH_2)_3$ (1.784 g, 6.32 mmol) with stirring. The red solution turned yellow-orange. After 21 h, the solvent was removed by oil pump vacuum. The residue was passed through a bed of silica gel (4.5 × 8.5 cm) using hexanes and then 2:1 v/v hexanes/CH_2Cl_2. The solvent was removed from the product-containing fractions by oil pump vacuum to give *trans*-$Fe(CO)_3(As((CH_2)_3CH{=}CH_2)_3)_2$ as a yellow oil (1.322 g, 1.876 mmol, 62%). Anal. Calcd for $C_{33}H_{54}As_2FeO_3$ (704.48): C 56.26, H 7.73. Found: C 56.19, H 7.79.

NMR ($CDCl_3$, δ/ppm): ^1H (500 MHz) 5.81 (ddt, $^3J_{HHtrans}$ = 16.9 Hz, $^3J_{HHcis}$ = 10.2 Hz, $^3J_{HH}$ = 6.6 Hz, 6H), 5.09-4.97 (m, 12H), 2.20-2.13 (m, 12H), 1.88-1.77 (m, 12H), 1.72-1.65, (m, 12H); $^{13}C\{^1H\}$ (125 MHz) 216.5 (s, CO), 138.0 (s, **C**H=), 115.4 (s, =**C**H_2), 35.5 (s, $AsCH_2CH_2$**C**-H_2), 26.9 (s, $AsCH_2$), 23.9 (s, $AsCH_2$**C**H_2). IR (oil film, cm^{-1}): 2928 (w), 2007 (m), 1936 (m),

1909 (m), 1852 (s, $\nu_{C\equiv O}$), 1641 (m), 1437 (w), 988 (m), 910 (m), 720 (w), 635 (s).

$Sb((CH_2)_6CH{=}CH_2)_3$. A flame dried Schlenk flask was charged with magnesium turnings (0.923 g, 38.0 mmol), THF (40 mL), and 1,2-dibromoethane (0.30 mL, 3.5 mmol) and was cooled to 0 °C. Then $Br(CH_2)_6CH{=}CH_2$ (6.0 g, 5.27 mL, 31.4 mmol) was added dropwise with stirring over 10 min. After 1 h, the cooling bath was removed. After 16 h, the remaining magnesium was removed by filtration. Following a standard protocol,[s1] an aliquot of the filtrate was used to determine the concentration of the Grignard reagent $BrMg(CH_2)_6CH{=}CH_2$. The filtrate was cooled to 0 °C, and $SbCl_3$ (2.15 g, 9.4 mmol; 1:3 Sb/RMgBr) in THF (10 mL) was added dropwise with stirring over 10 min. The cold bath was removed. After 15 h, the mixture was cooled to 0 °C and aqueous NH_4Cl (10 mL) was added dropwise with stirring over 10 min. After 1 h, the cold bath was removed. After 1 h, the aqueous phase was removed via syringe. The solvent was removed from the organic phase by oil pump vacuum, and CH_2Cl_2 (20 mL) was added. The solution was passed through a short pad of silica (2.5 × 5.0 cm), which was washed with CH_2Cl_2. The solvent was removed from the filtrate by oil pump vacuum to give $Sb((CH_2)_6CH{=}CH_2)_3$ as a colorless oil (2.28 g, 5.01 mmol, 53%). Anal. Calcd. For $C_{24}H_{45}Sb$ (455.38): C, 63.30; H, 9.96. Found: C, 62.84; H, 10.33.

NMR ($CDCl_3$, δ/ppm): ^1H (500 MHz) 5.82 (ddt, $^3J_{HHtrans}$ = 16.9 Hz, $^3J_{HHcis}$ = 10.1 Hz, $^3J_{HH}$ = 6.6 Hz, 3H), 5.05-4.91 (m, 6H), 2.07-1.98 (m, 6H), 1.49-1.18 (m, 30H); $^{13}C\{^1H\}$ (125 MHz) 139.3 (s, $\underline{C}H{=}$), 114.1 (s, $={\underline{C}}H_2$), 33.8 (s, $\underline{C}H_2CH{=}CH_2$), 29.7 (s, $SbCH_2CH_2\underline{C}H_2$), 29.6 (s, CH_2), 29.5 (s, CH_2), 29.2 (s, $Sb\underline{C}H_2$), 29.0 (s, $SbCH_2\underline{C}H_2$). IR (oil film, cm^{-1}): 3076 (w), 2920 (s), 2851 (s), 1641 (m), 1464 (m), 991 (m), 906 (s).

$Bi((CH_2)_6CH{=}CH_2)_3$. A flame dried Schlenk flask was charged with magnesium turnings (0.923 g, 38.0 mmol), THF (40 mL), and 1,2-dibromoethane (0.30 mL, 3.5 mmol) and was cooled to 0 °C. Then $Br(CH_2)_6CH{=}CH_2$ (6.0 g, 5.27 mL, 31.4 mmol) was added dropwise with stirring over 10 min. After 1 h, the cooling bath was removed. After 16 h, the remaining magnesium was removed by filtration. Following a standard protocol,[s1] an aliquot of the filtrate was used to determine the concentration of the Grignard reagent $BrMg(CH_2)_6CH{=}CH_2$. The filtrate was cooled to

0 °C, and $BiCl_3$ (2.97 g, 9.4 mmol; 1:3 Bi/RMgBr) in THF (10 mL) was added dropwise with stirring over 10 min. The cold bath was removed. After 15 h, the mixture was cooled to 0 °C and aqueous NH_4Cl (10 mL) was added dropwise with stirring over 10 min. After 1 h, the cold bath was removed. After 1 h, the aqueous phase was removed via syringe. The solvent was removed from the organic phase by oil pump vacuum, and CH_2Cl_2 (20 mL) was added. The solution was passed through a short pad of silica (2.5 × 5.0 cm), which was washed with CH_2Cl_2. The solvent was removed from the filtrate by oil pump vacuum to give $Bi((CH_2)_6CH{=}CH_2)_3$ as a colorless oil (2.60 g, 4.79 mmol, 51%). Anal. Calcd. For $C_{24}H_{45}Bi$ (542.60): C, 53.13; H, 8.36. Found: C, 52.23; H, 8.24.[s3]

NMR ($CDCl_3$, δ/ppm): ^{1}H (500 MHz) 5.82 (ddt, $^3J_{HHtrans}$ = 16.9 Hz, $^3J_{HHcis}$ = 10.2 Hz, $^3J_{HH}$ = 6.7 Hz, 3H), 5.03-4.91 (m, 6H), 2.08-2.02 (m, 6H), 1.92-1.79 (m, 10H), 1.41-1.29 (m, 20H); $^{13}C\{^1H\}$ (125 MHz) 139.3 (s, $\underline{C}$H=), 114.3 (s, =$\underline{C}H_2$), 35.2 (s, $\underline{C}H_2CH{=}CH_2$), 34.0 (s, $BiCH_2CH_2\underline{C}H_2$), 29.1 (s, CH_2), 29.0 (s), 28.9 (s, $Bi\underline{C}H_2$), 18.2 (s, $BiCH_2\underline{C}H_2$). IR (oil film, cm^{-1}): 3078 (w), 2924 (s), 2853 (m), 1641 (m), 1458 (m), 1360 (m), 1057 (m), 991 (m), 908 (s).

***trans*-Fe(CO)$_3$(Sb((CH$_2$)$_6$CH=CH((CH$_2$)$_6$)$_3$Sb) (16c).** Smaller scale reaction. A Schlenk flask was charged with **15c** (0.250 g, 0.238 mmol) and CH_2Cl_2 (67 mL; the resulting solution is 0.0035 M in **15c**) and fitted with a condenser. A solution of Hoveyda-Grubbs' second generation catalyst was prepared (0.030 g (0.048 mmol, 20 mol %) in 5 mL of CH_2Cl_2). Half was added dropwise over 5 min. The sample was refluxed. After 18 h, the remaining catalyst was added. After another 72 h, the solvent was removed by oil pump vacuum. The residue was filtered through neutral alumina (2.5 × 5 cm) using 2:1 v/v hexanes/CH_2Cl_2. The solvent was removed from the filtrate by oil pump vacuum to give **16c** as a yellow wax that solidified over time under vacuum to a yellow powder (0.161 g, 0.166 mmol, 70%, mixture of *E*/*Z* isomers).

Crystallography

A. A hexanes/CH_2Cl_2 solution of *out,out*-**8a** was refrigerated and allowed to slowly concentrate (vial with skew cap). After 3 d, a colorless block-shaped crystal was collected. Data were acquired as outlined in Table s1. Cell parameters were obtained from 45 data frames taken at

widths of 1°. Integrated intensity information for each reflection was obtained by reduction of the data frames with APEX3.[s4] Data were corrected for Lorentz and polarization factors, and crystal decay and absorption effects (using SADABS[s5]). CheckCIF showed the unit cell contains solvent accessible voids of 39 Å^3. Efforts to mask the solvent(s) using Olex2 suggested the number of electrons in the voids to be zero. The solvent mask also gave no improvement on the reliability factors. The absence of additional symmetry and voids was confirmed using PLATON (ADDSYM).[s6] The structure was refined (weighted least squares refinement on F^2) to convergence.[s7,s8] Hydrogen atoms were placed in idealized positions and refined using a riding model. All non-hydrogen atoms were refined with anisotropic thermal parameters.

B. A hexanes solution of *in,in*-**8a** was refrigerated and allowed to slowly concentrate (vial with skew cap). After 10 d, colorless plates were collected. Data were acquired as outlined in Table s1. Cell parameters were determined from 45 data frames taken at widths of 1° and refined with 26190 reflections. Integrated intensity information for each reflection was obtained by reduction of the data frames with CrysAlisPro.[s9] Numerical absorption corrections were based on Gaussian integrations over a multifaceted crystal model. Empirical absorption corrections were performed using spherical harmonics, implemented in SCALE3 ABSPACK scaling algorithm. Systematic reflection conditions and statistical tests indicated the compound crystallized in monoclinic *I*2/*a*, which was confirmed by ShelXT 2018/2.[s10] Elongated thermal ellipsoids and residual electron density peaks near C3-C5 and C7-C11 indicated possible disorder, which was modeled between two positions with occupancy ratios of 52:48 and 65:35, respectively. Restraints were used to keep the bond distances and thermal ellipsoids meaningful. The absence of additional symmetry was confirmed using PLATON.[s6] The structure was refined (weighted least squares refinement on F^2) to convergence.[s7,s8] Hydrogen atoms were placed in idealized positions and refined using a riding model. All non-hydrogen atoms were refined with anisotropic thermal parameters. The molecular structure exhibited a C_2 symmetry axis.

C.[s11] A hexanes solution of **8b** was refrigerated and allowed to slowly concentrate (vial with skew cap). After 3 d, tan blocks were collected. Data were acquired as outlined in Table s1.

Cell parameters were determined from 45 data frames taken at widths of 1° and refined with 6586 reflections. Integrated intensity information for each reflection was obtained by reduction of the data frames with APEX3.[s4] Data were corrected for Lorentz and polarization factors, and crystal decay and absorption effects (using SADABS).[s5] Hydrogen atoms were placed in idealized positions and refined using a riding model. All non-hydrogen atoms were refined with anisotropic thermal parameters. The absence of additional symmetry was confirmed using PLATON.[s6] The structure was refined (weighted least squares refinement on F^2) to convergence.[s7,s8]

D. A hexanes solution of **8c** was refrigerated and allowed to slowly concentrate (vial with skew cap). After 2 d, colorless, block-shaped prisms were collected. Data were acquired as outlined in Table s1. The structure was refined and solved as described in **C** for **8b**.

E.[s11] Diethyl ether was added to a small quantity of **8c**·2O, and CH_2Cl_2 was added until the **8c**·2O dissolved. The sample was refrigerated and allowed to slowly concentrate (vial with skew cap). After 21 d, a twinned colorless block was collected. Data were acquired as outlined in Table s1. Cell parameters were determined from 45 data frames taken at widths of 1°. CELL_NOW was used to identify a 180° rotational twin. Efforts were made to resolve overlap by collecting data at a crystal/detector distance of 120 mm. Trials were made to integrate the data with both twins. Application of TWINABS (for absorption corrections) followed by refinement gave a completeness below 90%, with many reflections discarded (by SAINT in APEX3 or TWINABS).[s12] Thus, only the major component of the twin was used. TWINROTMAT in PLATON[s6] showed the twin matrix, which closely matched that from CELL_NOW. The former was used for the final refinement. Integrated intensity information for each reflection was obtained by reduction of the data frames with APEX3.[s4] Data were corrected for Lorentz and polarization factors, and crystal decay and absorption effects (using SADABS).[s5] Four molecules of water were present for every molecule of **8c**·2O. Hydrogen atoms were placed in idealized positions and were refined using a riding model. While some of the water hydrogen atoms could be located from difference Fourier maps, others were fit into a hydrogen bonding motif. Thus, this aspect of the structure should not be further analyzed. All non-hydrogen atoms were refined with anisotropic thermal parameters. The

absence of additional symmetry or voids was confirmed using PLATON. The structure was refined (weighted least squares refinement on F^2) to convergence[s7,s8] and a completeness of 98.5%.

F. Methanol was added to a concentrated diethyl ether solution of **11c** until the sample became opaque. Then diethyl ether was added until the sample was again homogeneous. The capped solution was refrigerated. After 5 d, yellow blocks were collected. Data were acquired as outlined in Table s1. Cell parameters were determined from 45 data frames taken at widths of 1° and refined with 8637 reflections. Integrated intensity information for each reflection was obtained by reduction of the data frames using APEX3.[s4] Data were corrected for Lorentz and polarization factors, and crystal decay and absorption effects (using SADABS).[s5] Hydrogen atoms were placed in idealized positions and refined using a riding model. All non-hydrogen atoms were refined with anisotropic thermal parameters. Elongated ellipsoids on C32 and C33 suggested disorder, which was modeled between two positions with an occupancy ratio of 65:35. Restraints were added to keep the bond distances and thermal ellipsoids meaningful. The absence of additional symmetry or voids were confirmed using PLATON.[s6] The structure was refined (weighted least squares refinement on F^2) to convergence.[s7,s8]

G.[s11] Methanol was added to a concentrated diethyl ether solution of **12c** until the sample became opaque The diethyl ether was added until the sample was again homogeneous. The capped solution was refrigerated. After 3 d, a yellow plate was collected. Data were acquired as outlined in Table s1. Cell parameters were obtained from 45 data frames were taken at widths of 1°. Integrated intensity information for each reflection was obtained by reduction of the data frames using APEX3.[s4] Data were corrected for Lorentz and polarization factors, and crystal decay and absorption effects (using SADABS).[s5] Hydrogen atoms were placed in idealized positions and refined using a riding model. All non-hydrogen atoms were refined with anisotropic thermal parameters. The absence of additional symmetry was confirmed using PLATON (ADDSYM).[s6] Elongated ellipsoids and nearby residual electron density peaks close to C32F-C35F and Cl1D suggested disorder, which was modeled between two positions with an occupancy ratio of 59:41. Restraints were added to keep the bond distances and thermal ellipsoids meaningful. CheckCIF showed that

the unit cell contained voids of 33 Å^3. However, no residual electron density was detected, including masking efforts using Olex2. The structure was refined (weighted least squares refinement on F^2) to convergence.[s7,s8]

■ REFERENCES (continued)

(s1) Krasovskiy, A.; Knochel, P. Convenient Titration Method for Organometallic Zinc, Magnesium, and Lanthanide Reagents. *Synthesis* **2006**, *5*, 890−891.

(s2) Howell, J. A. S.; Johnson, B. F. G.; Josty, P. L.; Lewis, J. Synthesis and reactions of tetracarbonyl- and tricarbonyliron complexes of α,ß-unsaturated ketones. *J. Organomet. Chem.* **1972**, *39*, 329-333.

(s3) See Gabbaï, F. P.; Chirik, P. J.; Fogg, D. E.; Meyer, K.; Mindiola, D. J.; Schafer, L. L.; You, S.-L. An Editorial About Elemental Analysis. *Organometallics* **2016**, *35*, 3255-3256.

(s4) *APEX3*, *Program for Data Collection on Area Detectors*; BRUKER AXS Inc., 5465 East Cheryl Parkway, Madison, WI 53711-5373 USA.

(s5) Sheldrick, G. M. *SADABS, Program for Absorption Correction of Area Detector Frames*; BRUKER AXS Inc., 5465 East Cheryl Parkway, Madison, WI 53711-5373 USA.

(s6) Spek, A. L. Single-crystal structure validation with the program *PLATON*. *J. Appl. Cryst.* **2003**, *36,* 7-13.

(s7) Dolomanov, O. V; Bourhis, L. J.; Gildea, R. J.; Howard, J. A. K.; Puschmann, H. *OLEX2*: A Complete Structure Solution, Refinement and Analysis Program. *J. Appl. Crystallogr.* **2009**, *42*, 339-341.

(s8) (a) Sheldrick, G. M. A short history of *SHELX*. *Acta Crystallogr., Sect. A: Found. Crystallogr.* **2008**, *64*, 112-122. (b) Sheldrick, G. M. Crystal structure refinement with *SHELXL*. *Acta Cryst*. **2015**, *C71*, 3-8.

(s9) *CrysAlisPRO* Software System; Rigaku Oxford Diffraction/Agilent Technologies UK Ltd, Yarnton, England (2023).

(s10) Sheldrick, G. M. *SHELXT* – Integrated space-group and crystal-structure determination. *Acta Crystallogr., Sect. A: Found. Adv.* **2015**, *71*, 3-8.

(s11) This structure solution and attendant data are repeated from our preliminary communication: Zarcone, S. R.; Verardi, P. J.; Bhuvanesh, N.; Gladysz, J. A. A Surprise Landing on the *Terra Incognita* of Macrocyclic Dibridgehead Diorganoarsines: Syntheses, Structures, and Reactivities. *Chem. Commun.* **2022**, *58*, 8694-8697.

(s12) *SAINT (Version 7), Program for Data Integration from Area Detector Frames*; Bruker AXS Inc., Madison, WI 53711-5373 USA.

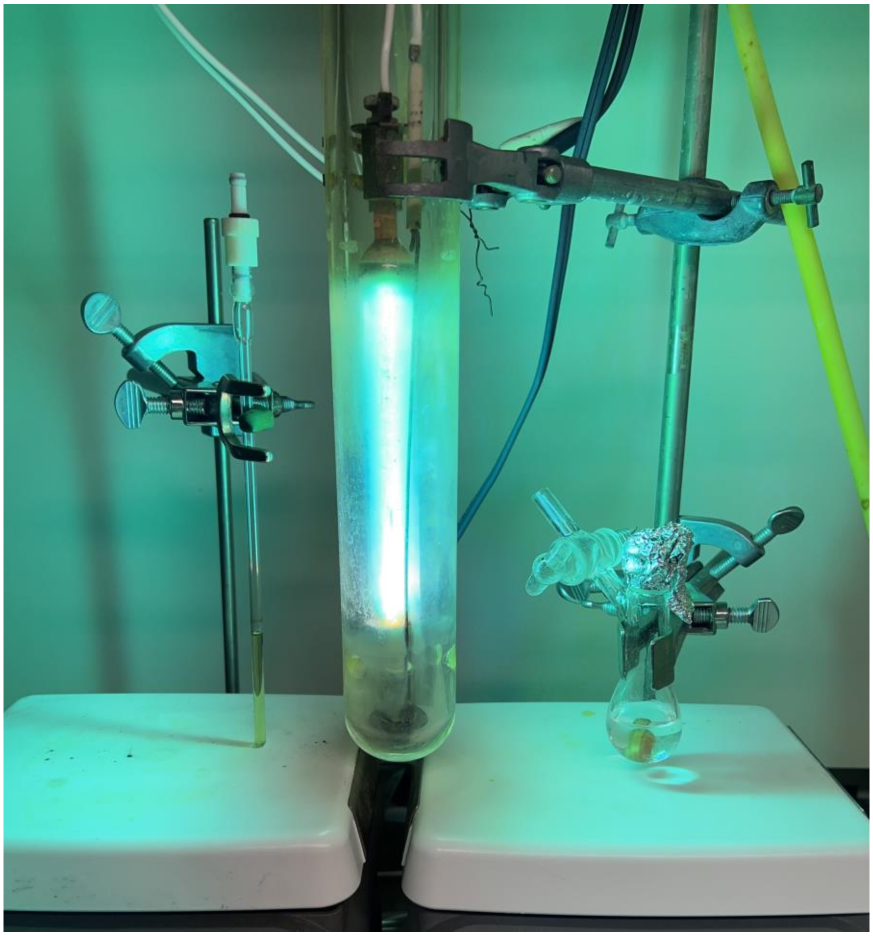

Figure s1. Conditions for the photochemical demetallations of **7a-c** to **8a-c** in Scheme 5 and **18c** to **20c** in Scheme 8; reactions can be conducted on NMR tube or Schlenk flask scales. **CAUTION**: the 450 W lamp emits considerable heat, and interruption of water cooling (e.g., flow outage due to low pressure or hose detachment due to high pressure or a failed juncture) can lead to glass failure, solvent ignition, explosions, or worse.

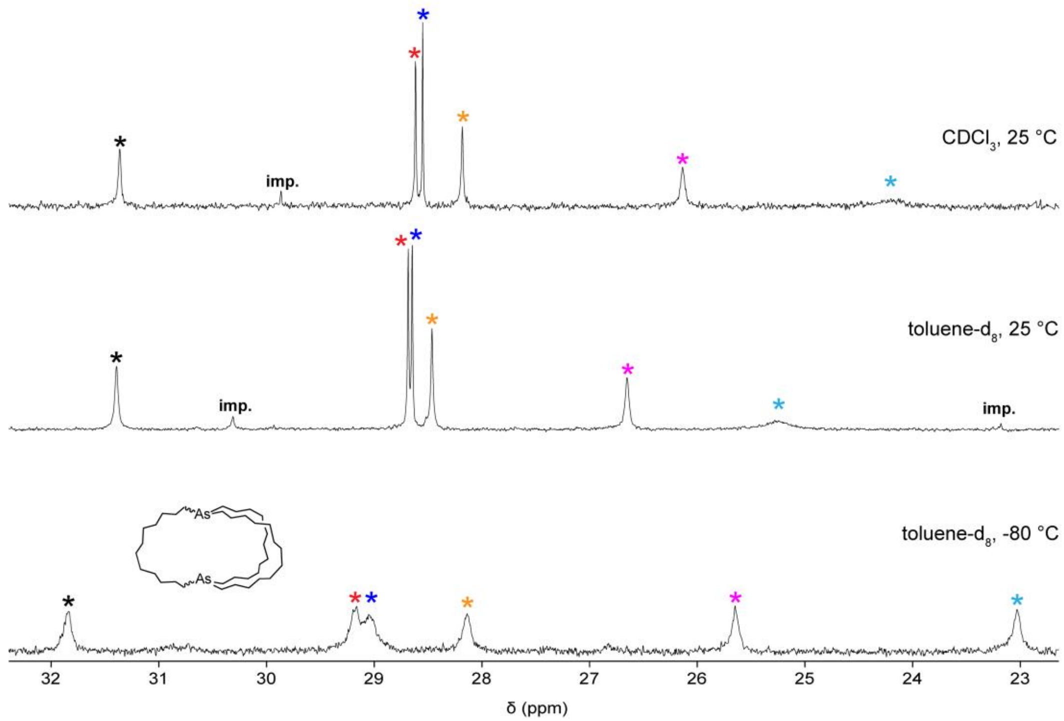

Figure s2. $^{13}C\{^{1}H\}$ NMR spectra (125 MHz) of **8b** in either $CDCl_3$ at 25 °C or toluene-d_8 as a function of temperature. Impurities are denoted by "imp."

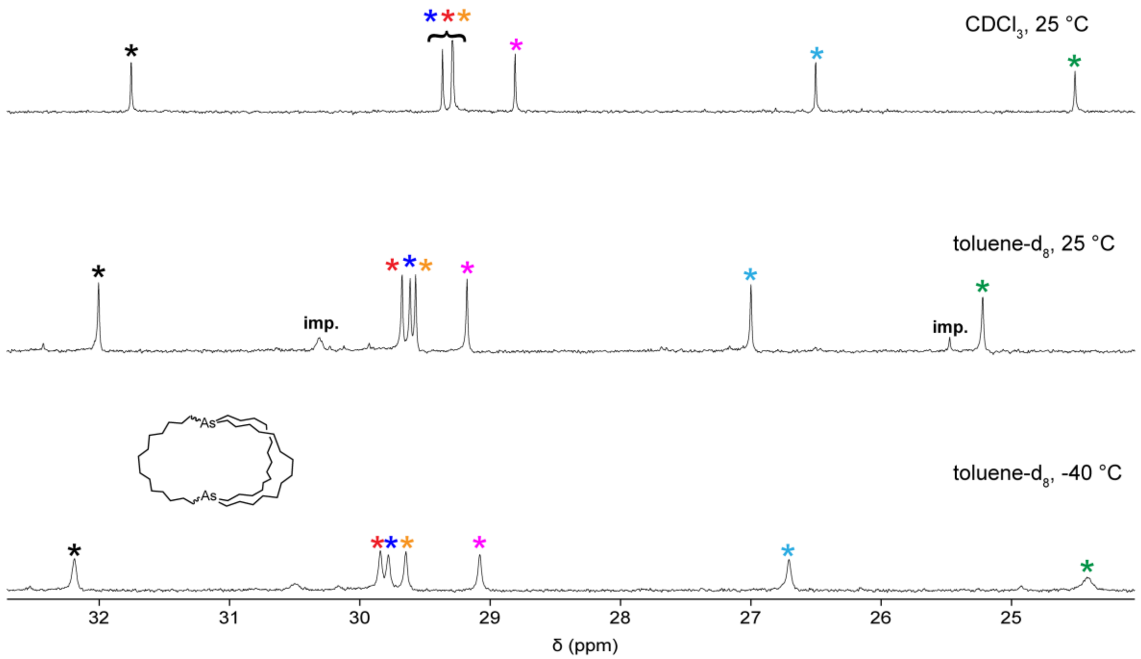

Figure s3. $^{13}C\{^{1}H\}$ NMR spectra (125 MHz) of **8c** in either $CDCl_3$ at 25 °C or toluene-d_8 as a function of temperature. Impurities are denoted by "imp.".

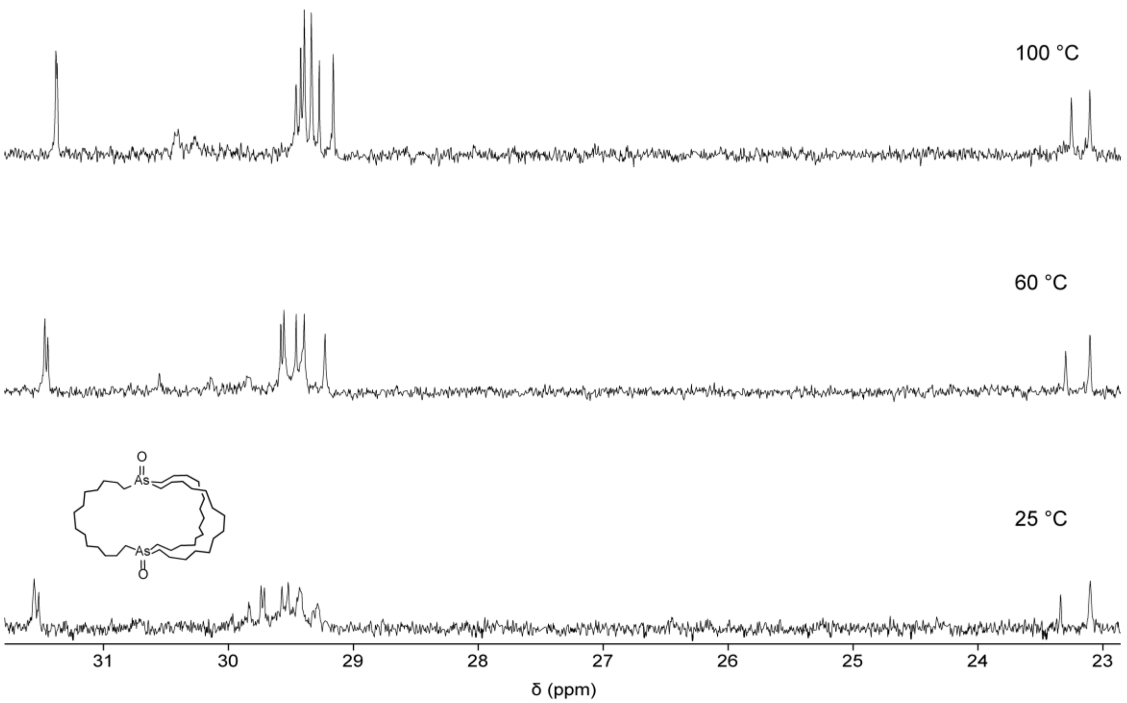

Figure s4. $^{13}C\{^1H\}$ NMR spectra (125 MHz) of **8c**·2O in C_6D_5Br as a function of temperature.

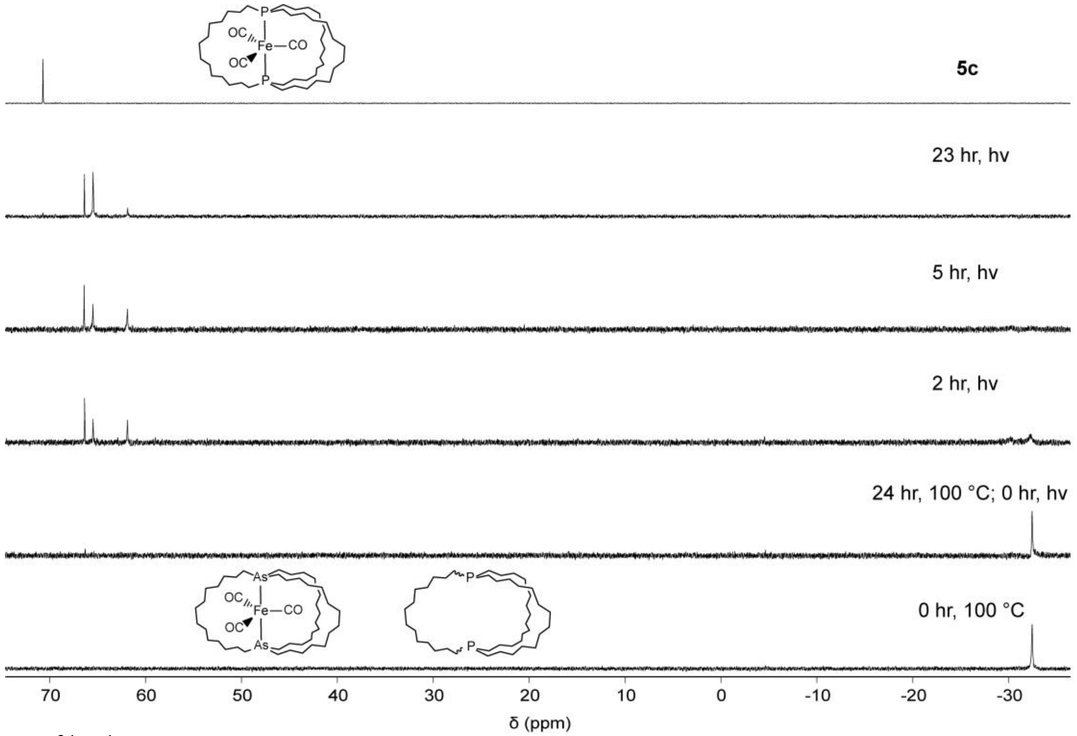

Figure s5. $^{31}P\{^1H\}$ NMR spectra (202 MHz) of **1c** and **7c** in toluene-d_8 at 100 °C and then during photolysis at RT; see Scheme 7 (bottom) and Figure s1. A spectrum of **5c** in toluene-d_8 at RT (top) is included for reference.

Table s1. Summary of crystallographic data.

	in,in-**8a**	*out,out*-**8a**	*out,out*-**8b**	*out,out*-**8c**	*out,out*-**8c**·2O·$(H_2O)_4$	**11c**	**12c**
empirical formula	$C_{30}H_{60}As_2$	$C_{30}H_{60}As_2$	$C_{36}H_{72}As_2$	$C_{42}H_{84}As_2$	$C_{42}H_{92}As_2O_6$	$C_{42}H_{84}As_2$ Cl_2Pt	$C_{42}H_{84}As_2$ Cl_2Pd
formula weight	570.62	570.62	654.77	738.93	842.99	1004.92	916.23
temperature [K]	100.00	110.00	110.00	110.00	110.00	110.00	110.00
diffractometer	XtaLAB Synergy, Dualflex, HyPix	BRUKER Quest	Bruker Venture	Bruker Venture	Bruker Venture	Bruker Venture	BRUKER Quest
wavelength [Å]	1.54184	0.71073	1.54178	1.54178	1.54178	1.54178	0.71073
crystal system	Monoclinic	Trigonal	Triclinic	Trigonal	Triclinic	Monoclinic	Triclinic
space group	*I*2/*a*	*R*-3*c*	*P*-1	*R*-3*c*	*P*-1	$P2_1$/n	*P*-1
unit cell dimens:							
a [Å]	17.4043(2)	9.2894(4)	9.2624(6)	9.2595(7)	8.6319(6)	16.3117(5)	17.6069(8)
b [Å]	10.5784(2)	9.2894(4)	9.3808(6)	9.2595(7)	13.4245(10)	13.9045(5)	17.7675(8)
c [Å]	17.4236(2)	63.279(3)	25.6699(17)	90.185(10)	20.7781(15)	20.1180(6)	30.5063(14)
α [°]	90	90	85.931(2)	90	72.297(2)	90	105.1660(10)
β [°]	104.7850(10)	90	83.106(2)	90	84.548(2)	91.2943(13)	97.5980(10)
γ [°]	90	120	61.125(2)	120	88.073(2)	90	90.6490(10)
V [$Å^3$]	3101.64(8)	4729.0(5)	1938.8(2)	6696.4(13)	2283.3(3)	4561.7(3)	9119.5(7)
Z	4	6	2	6	2	4	8
Z'	0.5	0.167	1	0.167	1	1	4
ρcalc[Mg/m^3]	1.222	1.202	1.122	1.099	1.226	1.463	1.335
μ[mm^{-1}]	2.749	2.134	2.255	2.008	2.123	8.619	1.991
F(000)	1224	1836	708	2412	916	2056	3856
crystal size [mm^3]	0.18 × 0.14 × 0.02	0.40 × 0.38 × 0.12	0.06 × 0.01 × 0.01	0.38 × 0.14 × 0.07	0.16 × 0.12 × 0.08	0.04 × 0.03 × 0.03	0.20 × 0.18 × 0.05
θ limit[°]	4.937 to 70.030	2.612 to 30.491	3.469 to 65.585	5.603 to 70.013	3.456 to 65.565	3.527 to 69.989	1.691 to 27.561
index range (h, k, l)	–22/22, –11/ 13, –22/22	–13/13, –13/13, –90/90	–10/10, –11/11, –30/30	–9/11, –11/11, –106/109	–10/9, –15/15, –24/24	–19/19, –16/15, –24/24	–22/22, –23/23, –39/39
reflections collected	26190	30320	26789	7195	30552	59120	520407
indep. reflections	2928	1609	6586	1422	7756	8637	42015
R(int)	0.0286	0.0321	0.0347	0.0270	0.0545	0.0510	0.0560
completeness to θ	99.4	99.4	98.7	99.3	98.5	100	99.9
max. and min. transmission	1.000 and 0.462	0.4330 and 0.2678	0.3795 and 0.2800	0.322 and 0.207	0.4649 and 0.3677	0.3520 and 0.1685	0.4305 and 0.3450
data/restraints/ parameters	2928 / 298 / 219	1609 / 0 / 49	6586 / 0 / 343	1422 / 0 / 67	7756 / 0 / 458	8637 / 97 / 443	42015 / 314 / 1739
goodness-of-fit, F^2	1.078	1.139	1.048	1.127	1.169	1.060	1.065
R indices (final) [I> 2σ(I)]							
R_1	0.0418	0.0253	0.0314	0.0304	0.0678	0.0265	0.0258
wR_2	0.1129	0.0612	0.0855	0.0782	0.1677	0.0669	0.0609
R indices (all data)							

R_1	0.0451	0.0272	0.0341	0.0311	0.0717	0.0288	0.0320
wR_2	0.1165	0.0620	0.0874	0.0788	0.1707	0.0688	0.0624
largest diff. peak and hole [e$Å^{-3}$]	0.580, -0.592	1.223, -0.434	0.949, -0.398	1.294, -0.415	1.14, -0.609	1.618, -0.748	1.639, -1.713

Table s2. Key crystallographic distances [Å] and angles [°] for platinum and palladium complexes **11c** and **12c**.

	**11c**	**12c**(1)[a]	**12c**(2)[a]	**12c**(3)[a]	**12c**(4)[a]
M–As	2.3792(3)<br>2.3837(3)	2.4006(2)<br>2.4038(2)	2.4058(2)<br>2.4079(2)	2.3947(3)<br>2.3984(2)	2.3946(2)<br>2.4067(2)
As···As	4.7601(5)[b]	4.8014(6)[c]	4.8114(7)	4.7929(6)	4.7954(5)
As–C	1.943(3)<br>1.941(3)<br>1.943(3)<br>1.945(3)<br>1.938(3)<br>1.948(3)	1.9450(19)<br>1.9470(18)<br>1.9485(19)<br>1.9470(18)<br>1.9433(18)<br>1.9467(18)	1.9477(19)<br>1.9505(18)<br>1.9417(19)<br>1.9488(19)<br>1.9508(19)<br>1.9545(19)	1.932(2)<br>1.9510(19)<br>1.954(2)<br>1.943(2)<br>1.952(2)<br>1.9365(19)	1.9393(19)<br>1.9471(18)<br>1.9452(18)<br>1.9522(19)<br>1.9467(19)<br>1.9532(19)
M–Cl	2.2984(8)<br>2.3089(7)	2.2915(5)<br>2.2965(5)	2.3007(5)<br>2.2909(5)	2.315(3)<br>2.2860(5)	2.3086(5)<br>2.3063(5)
As–M–As	176.183(11)	175.911(9)	176.650(9)	178.998(11)	174.352(8)
C–As–M	114.23(9)<br>112.52(9)<br>114.44(10)<br>109.91(9)<br>115.45(9)<br>112.61(9)	110.66(6)<br>119.42(6)<br>115.16(6)<br>111.77(6)<br>108.28(6)<br>118.11(6)	109.42(6)<br>118.79(6)<br>113.90(6)<br>111.76(6)<br>120.73(6)<br>111.72(6)	110.54(6)<br>119.24(6)<br>111.94(6)<br>111.29(6)<br>116.55(6)<br>114.05(6)	110.95(6)<br>115.69(6)<br>117.06(6)<br>112.16(6)<br>124.22(6)<br>111.31(6)
Cl–M–Cl	178.71(3)	178.37(2)	179.47(3)	172.79(11)	178.45(2)
As–M–Cl	89.34(2)<br>85.81(2)<br>93.06(2)<br>91.82(2)	89.920(14)<br>92.832(14)<br>90.580(14)<br>86.752(14)	92.588(15)<br>85.345(15)<br>87.246(14)<br>94.797(14)	86.78(12)<br>92.43(12)<br>93.236(14)<br>87.466(14)	87.283(13)<br>87.257(13)<br>92.677(13)<br>92.821(13)
C–As–C	105.06(13)<br>104.69(13)<br>104.93(13)<br>105.41(13)<br>106.84(12)<br>106.03(14)	100.18(8)<br>104.48(9)<br>104.99(8)<br>103.45(8)<br>107.92(8)<br>106.28(8)	100.48(8)<br>107.95(8)<br>105.10(8)<br>104.05(9)<br>101.39(8)<br>105.20(8)	105.55(9)<br>103.14(9)<br>105.03(9)<br>101.21(9)<br>106.77(9)<br>105.80(9)	106.07(8)<br>100.53(8)<br>104.98(8)<br>100.16(8)<br>106.20(9)<br>99.60(8)

[a]Values for the four independent molecules in the unit cell. [b]Phosphorus-phosphorus distance in the corresponding adduct of **1c** and $PtCl_2$: 4.611 Å. [c]Phosphorus-phosphorus distance in the corresponding adduct of **1c** and $PdCl_2$: 4.630 Å.
